# Genetic alterations in poor-quality individually selected sperm highlight candidate biomarkers for male subfertility

**DOI:** 10.1038/s41598-026-50620-0

**Published:** 2026-04-28

**Authors:** Mohammad A. Al Smadi, Aftab Ali Shah, Muhammad Riaz Khan, Eman Alshdaifat, Ulrike Fischer, Hashim Abdul-Khaliq, Eckart Meese, Masood Abu-Halima

**Affiliations:** 1 Department of Medical Laboratory Science, Al Al-Bayt University, Mafraq, Jordan; 2 Department of Biotechnology, Faculty of Biological Sciences, University of Malakand, Chakdara, Khyber Pakhtunkhwa Pakistan; 3 Department of Obstetrics and Gynecology, Faculty of Medicine, Yarmouk University, Irbid, Jordan; 4 Institute of Human Genetics, Saarland University, 66421 Homburg, Germany; 5 Department of Paediatric Cardiology, Saarland University Hospital, Homburg, Germany

**Keywords:** Male subfertility, Whole-genome sequencing, Sperm quality, Candidate variants, Genomic instability, Genetics, Molecular biology, Urology

## Abstract

Whole-genome sequencing (WGS) of morphology-selected sperm pools provides an exploratory approach for investigating genomic features associated with variable sperm quality. In this study, pooled DNA from 1500 individually selected spermatozoa per sample was analyzed from paired high-quality (HQ; motile, normal morphology) and poor-quality (PQ; immotile, abnormal morphology) sperm populations obtained from the same ejaculate of six male partners undergoing infertility treatment. A multi-step filtering and annotation pipeline incorporating American College of Medical Genetics and Genomics (ACMG) criteria, population allele frequencies, tissue-specific expression, and predicted protein effects identified 42 unique high-confidence variants affecting 32 genes across all samples, all consistent with homozygous genotypes in the corresponding individuals as inferred from pooled sperm DNA. Across descriptive comparisons, PQ sperm pools frequently showed numerically higher counts across several variant categories, including structural alterations, splice-related annotations, and coding variant annotations, although none of these differences reached statistical significance. Because HQ and PQ pools were derived from the same individuals, these observations were interpreted descriptively rather than as evidence of distinct inherited variant burden between sperm quality groups. A frameshift insertion in *FOXO6*, detected in a single PQ sample, represented the most notable exploratory candidate variant, while recurrent variants such as *NPIPB15* p.Tyr301Cys and *ANKRD36C* p.Gly311Asp were observed across multiple samples. When considered together with semen characteristics and pregnancy outcomes, the data support the possibility that poor-quality sperm may be associated with altered genomic integrity, particularly at the level of structural instability, while also highlighting recurrent candidate variants for further study. These findings support the value of broad genomic profiling of morphology-selected sperm pools as an exploratory strategy for investigating male subfertility and identifying candidate variants for future validation.

**Supplementary Information:**

The online version contains supplementary material available at 10.1038/s41598-026-50620-0.

## Introduction

Male infertility, affecting approximately 7% of the global male population, represents a significant health concern and accounts for nearly half of all infertility cases worldwide^1^. This condition involves the complex interaction of genetic factors and lifestyle components. However, the extent of unexplained factors can reach up to 40% without the existence of an identifiable genetic cause^1–3^. Traditional diagnostic semen analysis has served as the clinical basis for evaluating male infertility. While this traditional method offers valuable insights into the evaluation of sperm function, it has failed to identify the underlying genetic contributors that frequently influence sperm dysfunction. Over the past decades, a substantial decline in semen quality has been reported, commonly attributed to environmental and lifestyle factors; however, the precise mechanisms driving this decline remain unclear^4^.

In recent years, genetic methodologies have been increasingly integrated into conventional semen evaluation for the diagnosis of male infertility. Techniques such as karyotyping, Y-chromosome microdeletion analysis, DNA fragmentation assessment, biomarker-based testing of specific genes implicated in infertility, and RNA profiling are now commonly employed^5–10^. These advances have improved the understanding of the genetic aspects of the condition. However, even men with normozoospermia may exhibit significant sperm DNA damage, suggesting that current WHO reference limits may lack sufficient sensitivity to predict fertility potential^11^. Moreover, traditional genetic methods often overlook subtle chromosomal rearrangements, minor deletions, or point mutations, while the biological and clinical implications of sperm DNA fragmentation remain controversial^7^. These limitations underscore the need for more comprehensive and sensitive diagnostic approaches capable of detecting rare or subtle genetic abnormalities that contribute to the estimated 40–50% of unexplained male infertility cases^1^.

The advent of genomic technologies such as whole-genome sequencing (WGS) presents promising opportunities for identifying genetic variants that impact the functionality of sperm cells^5,8,9,12–14^. WGS identifies diverse genetic variants across the whole genome, enabling comprehensive variant detection, and this could help in identifying novel biomarkers and deciphering the genetic causes of male infertility^8,14–16^. Additionally, the use of variant-filtering strategies that prioritize pathogenic and likely pathogenic variants significantly enhances the clinical utility of WGS, especially when applied to sperm-derived DNA^8^. This approach enables the identification of genetic variants that elicit functional changes directly affecting the biological efficiency of the involved gene(s)^17^. The WGS approach has already been efficient in the identification of mutations contributing to problems of sperm impairments^8,17^. These insights highlight the potential of WGS to improve diagnostic precision for variants in individuals undergoing assisted reproductive technologies (ART).

In addition to detecting known mutations, WGS has enabled the identification of monogenic causes of male infertility, implicating genes involved in meiosis, acrosome biogenesis, flagellar assembly, and testicular development^18,19^. Integrating WGS with traditional semen analysis may therefore provide a more comprehensive and accurate diagnostic platform. Advances in single-cell RNA sequencing (scRNA-seq) further support the value of genomic approaches^20^. For example, sperm selected for intracytoplasmic sperm injection (ICSI) from men with severe oligoteratozoospermia showed a significantly higher aneuploidy rate even when morphologically normal^21^. Additionally, other high-throughput platforms, such as single sperm sequencing, have similarly revealed substantial intra-ejaculate genetic heterogeneity, reinforcing the relevance of single-sperm sequencing for fertility diagnostics^5,22,23^. Although technical challenges remain, recent advances in cell isolation, whole-genome amplification, and sequencing technologies have greatly improved the feasibility of single-sperm sequencing for research and potential clinical diagnostic applications^20–22,24^.

In this study, WGS was performed on pooled DNA derived from individually selected spermatozoa collected from subfertile men undergoing infertility treatment. Sperm were selected according to morphology and motility, generating paired high-quality (HQ) and poor-quality (PQ) sperm pools from the same ejaculate in each participant. A total of 12 pooled sperm samples were analyzed: six HQ pools with normal morphology and motility and six PQ pools with reduced motility and abnormal morphology. Variants predicted to affect protein structure or function were identified and evaluated in the context of sperm-related gene expression and IVF outcomes. By integrating genomic profiling with semen characteristics and reproductive outcomes, this study aimed to explore candidate variants and genomic patterns potentially associated with impaired sperm quality and male subfertility.

## Materials and methods

### Subjects of study

The study a cohort comprised of six male participants recruited from couples attending an infertility clinic, all of whom had experienced failure to conceive following after at least 12 months of regular, unprotected sexual intercourse. These patients fulfilled the criteria for male subfertility as defined by the World Health Organization (WHO) 2010 guidelines. The WHO 2010 reference framework was adopted to ensure methodological consistency with the clinical semen analysis protocols applied during the study period and to enable standardized classification of sperm parameters across the cohort. Inclusion criteria included failure to achieve conception after 12 months of unprotected intercourse and semen characteristics consistent with WHO 2010 reference thresholds, including abnormalities in one or more conventional semen parameters, particularly progressive motility and/or sperm morphology (Supplemental Table 1). Sperm concentrations in the cohort ranged from 14 to 65 million/mL; therefore, indicating the absence of azoospermia or severe oligozoospermia within the cohort.

**Table 1:** Descriptive comparison of variant counts and annotations in high-quality (HQ) and poor-quality (PQ) sperm pools.

Category	Variant type	HQ (Mean ± SD)	PQ (Mean ± SD)	Cohen’s d
Overall variant counts	SNVs	3,293,840 ± 515,054	3,537,305 ± 141,139	− 0.65
	Deletions	348,967 ± 83,675	385,656 ± 39,050	− 0.56
	Insertions	338,408 ± 83,097	382,340 ± 41,943	− 0.67
	Sequence alterations	45,977 ± 16,628	52,133 ± 7922	− 0.47
Coding and protein-altering annotations	Frameshift variants	268 ± 61	290 ± 32	− 0.45
	Stop-gained variants	123 ± 24	132 ± 15	− 0.46
	Start-lost variants	32 ± 10	36 ± 5	− 0.54
	Protein-altering variants	2.2 ± 1.7	4.2 ± 2.3	− 0.98
	Missense variants	9260 ± 1993	10,063 ± 1207	− 0.49
	Synonymous variants	8398 ± 1981	9231 ± 1246	− 0.50
Splice-related and prediction-based annotations	Splice donor variants	312 ± 50	339 ± 30	− 0.63
	Splice acceptor variants	226 ± 42	244 ± 14	− 0.57
	SIFT: deleterious	1182 ± 223	1291 ± 137	− 0.59
	PolyPhen: probably damaging	494 ± 92	534 ± 67	− 0.50

Samples: high-quality (HQ; motile with normal morphology, n = 6) and poor-quality (PQ; immotile with abnormal morphology, n = 6) sperm pools.

SNV, single-nucleotide variant.

Data are presented as mean ± standard deviation (SD) for selected representative variant categories and annotations.

Cohen’s d is provided as a descriptive measure of effect size; negative values indicate lower counts in HQ pools than in PQ pools.

No comparisons reached statistical significance.

Because HQ and PQ pools were derived from the same individuals, these comparisons are presented descriptively and are not interpreted as evidence of distinct inherited variant burden between groups.

All participant provided written informed consent, and the study protocol received approval from the relevant Institutional Review Board. Female partner hormonal parameters and IVF outcome data were recorded to provide contextual support; however, the current study was not designed to discriminate between isolated male-factor infertility and combined male–female factor infertility. Information on karyotype abnormalities, Y-chromosome microdeletions, obstructive causes of infertility, and male hormonal parameters was not systematically available for all participants and therefore could not be uniformly incorporated into the present analysis.

### Semen analysis and sperm collection

**Fig. 1:**
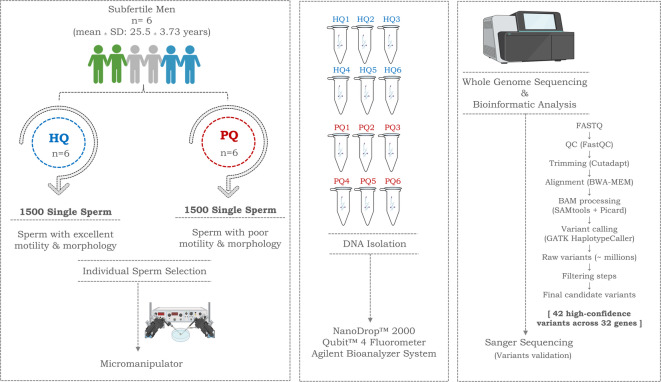
Flowchart of the study design.

Following the initial semen analysis, sperm samples were processed using PureSperm® density gradient centrifugation, the swim-up method, or a combination of both. The resulting sperm fractions were washed and transferred in a minimal volume of medium suitable for intracytoplasmic sperm injection (ICSI). Sperm selection was performed by trained embryologists using a micromanipulator system (CooperSurgical) equipped with a high-quality digital camera (Nikon Corporation). To minimize potential selection bias, embryologists were not informed of the downstream genomic analyses. Sperm were classified into two groups, as illustrated in Fig. 1:

- **High-quality sperm (HQ):** motile with normal morphology (n = 6)
- **Poor-quality sperm (PQ):** immotile with abnormal morphology (n = 6)

A total of 12 pooled sperm samples were analyzed (HQ, n = 6; PQ, n = 6). Each participant contributed one HQ pool and one PQ pool derived from the same ejaculate immediately after ICSI. For each pool, 1500 individually selected spermatozoa were collected and combined before DNA extraction, yielding a total of 18,000 selected spermatozoa across all samples.

### Isolation of DNA for DNA sequencing

DNA extraction from pooled, individually selected spermatozoa was performed using the QIAamp® DNA Micro Kit (Qiagen) on 12 samples (HQ, n = 6; PQ, n = 6), following the manufacturer’s protocol with minor modifications. Briefly, selected spermatozoa were washed twice with phosphate-buffered saline (PBS) and then incubated in 50 µL of Buffer X2 containing 20 mM Tris·Cl (pH 8.0), 20 mM EDTA, 200 mM NaCl, 80 mM DTT (freshly added), 4% SDS, and 250 μg/mL Proteinase K (freshly added) (Sigma-Aldrich). The mixture was incubated at 55°C for 1 h with periodic inversion every 15 min. After incubation, the extraction procedure was completed using the QIAcube™ robot (Qiagen) to ensure standardized processing conditions and minimize between-sample variability. The remaining steps were performed according to the manufacturer’s instructions. DNA quantity was measured using the Qubit™ 4 Fluorometer (Thermo Fisher Scientific).

DNA integrity was evaluated using the Agilent 2100 Bioanalyzer (Agilent Technologies), and concentration and purity were further assessed using a NanoDrop spectrophotometer (Thermo Fisher Scientific). Gel electrophoresis was additionally used to examine DNA integrity and fragment size. DNA samples were stored at − 20 °C until further analysis.

### DNA library preparation and sequencing

DNA libraries were prepared using the MGIEasy FS DNA Library Prep Kit (MGI Technologies). For each of the 12 pooled sperm DNA samples (HQ, n = 6; PQ, n = 6), library preparation included fragmentation, end repair, A-tailing, adapter ligation, and PCR amplification. Initially, 5 ng of DNA from each sample was processed according to the MGIEasy FS DNA Library Prep Manual (Version B4). Genomic DNA was fragmented using Frag Buffer II and Frag Enzyme II, followed by purification with DNA Clean Beads and quantification using the Qubit® dsDNA HS Assay Kit (Thermo Fisher Scientific). Fragmented products then subjected to end repair and A-tailing using ERAT Buffer and ERAT Enzyme Mix according to the manufacturer’s thermocycler program. MGIEasy DNA Adapters, Ligation Buffer, and DNA Ligase were subsequently added, and the ligation reaction was incubated at 23 °C for 30 min. The sample was then purified using DNA Clean Beads according to MGI guidelines.

PCR amplification was performed using PCR Enzyme Mix and PCR Primer Mix, with 18 amplification cycles, followed by purification of the resulting products using DNA Clean Beads. Purified PCR products were quantified using the Qubit® dsDNA HS Assay Kit (Thermo Fisher Scientific), and fragment size distribution was assessed using the High Sensitivity DNA Kit (Agilent Technologies). Subsequent steps included denaturation of PCR products at 95 °C for 3 min, followed by single-strand circularization using Splint Buffer and DNA Rapid Ligase on ice and incubation at 37 °C for 30 min. Post-circularization, enzymatic digestion of linear single-stranded products was carried out using Digestion Buffer and Digestion Enzyme at 37 °C for 30 min.

The resulting products were purified using DNA Clean Beads according to the MGI operating manual and sequenced on the MGI BGISEQ-G400 platform (MGI Technologies) according to the manufacturer’s instructions.

### Processing, mapping, filtering, and variant identification in DNA sequencing data

In DNA sequencing data analysis, following multiplexing and demultiplexing, raw data from different lanes sharing the same barcode were merged for further analysis. Before moving on to the next stages, the quality of the combined raw reads was evaluated using FastQC (Version 0.11.9; https://www.bioinformatics.babraham.ac.uk/projects/fastqc/) on the merged FASTQ files. To refine data quality, Cutadapt (Version 4.0; https://cutadapt.readthedocs.io/)^25^ was employed for trimming according to the specified parameters (quality cutoff of 30 for both 5′ and 3′ ends with -q 30,30, and a minimum read length of 20 bases after adapter removal with -m 20). Following trimming, a second round of FastQC checks confirmed the enhancement in sequence data quality. The raw sequencing reads were aligned to the GRCh38 reference genome using the Burrows–Wheeler Aligner (BWA-MEM, Version 0.7.17; http://bio-bwa.sourceforge.net/) algorithm^26^.

Before mapping, a reference genome index was created with the ‘bwa index’ command to enhance sequence search efficiency. Subsequently, the alignment of reads was performed using the ‘bwa mem’ command. The resulting alignment data in Sequence Alignment/Map (SAM) format was converted to sorted Binary Alignment/Map (BAM) format using the SAMtools suite (Version 1.16.1; http://www.htslib.org/)^27^. To evaluate the alignment and counts data quality, QualiMap 2.0 (Version 2.3; http://qualimap.conesalab.org/) was employed^28^. Duplicate sequences were identified and marked using Picard tools (Picard Toolkit, Version 2.27.4; Broad Institute; https://broadinstitute.github.io/picard/). Subsequently, the marked and header-enhanced BAM file was indexed using the ‘SAMtools index’ command^27^. Variant calling was conducted using the Genome Analysis Toolkit (GATK, Version 4.3.0.0; https://gatk.broadinstitute.org/)^29^. Before this, genome dictionary files were generated using the Picard-tools CreateSequenceDictionary command, and the reference genome was indexed with SAMtools faidx^27^.

The HaplotypeCaller function^29^ within GATK was then applied to generate variant call format (VCF) files from the pooled sperm DNA samples. As sequencing was performed on pooled DNA derived from 1500 individually selected spermatozoa per sample, variant calls were interpreted as reflecting the pooled germline signal from the corresponding individual rather than the genotype of individual spermatozoa.

Identified variants, including single-nucleotide polymorphisms (SNPs) and insertions/deletions (indels), were processed through rigorous filtering based on GATK standard criteria. SNPs were filtered based on quality, strand bias, mapping quality, strand odds ratio, and various rank sum tests. Indels, which encompass both insertions and deletions, were similarly filtered using specific criteria. Base quality scores were recalibrated using known sites from known variant sites and SNP databases, and the recalibrated data were applied back to the original BAM file.

The final mutation calling utilized the recalibrated BAM file with the HaplotypeCaller function. Further refinement of the variants involved applying SnpSift (Version 5.1; part of the SnpEff suite; http://snpeff.sourceforge.net/)^30^, a tool from the SnpEff software suite, which applied filters for quality (QUAL ≥ 30), mapping quality (MQ = 30), and depth (DP ≥ 10). As DNA was derived from pooled sperm samples rather than diploid somatic tissue, zygosity-based interpretation was restricted to the inference of variants consistent with homozygous genotypes in the corresponding individuals. These inferred genotypes were not interpreted as representing genotypes of individual sperm cells.

### Variant filtering and annotation

Following the initial processing of WGS data, the primary objective of the study was to identify genomic variants of potential biological and clinical relevance. A structured filtering workflow was applied to prioritize candidate variants for further investigation. For each gene harboring candidate variants, variants were filtered based on the following criteria: genomic region (exonic or splice-related), read depth (≥ 20), gnomAD frequency (< 1%), American College of Medical Genetics and Genomics (ACMG) classification (VUS, pathogenic, or likely pathogenic), mode of inheritance (autosomal recessive or X-linked), and effect (e.g., nonsynonymous, frameshift, splice donor, splice acceptor, start-loss, stop-gain, or in-frame variant). Variants that fulfilled the above criteria were highlighted for further investigation.

The WGS data were initially uploaded to the Franklin platform (https://franklin.genoox.com/clinical-db/home) for automated variant interpretation. The initial filtering was performed using seven rigorous criteria. First, variants were filtered based on pathogenicity, with categories including pathogenic (P), likely pathogenic (LP), uncertain (leaning pathogenic), and variant of uncertain significance (VUS), while variants classified as leaning benign, likely benign, or benign were excluded from further analysis.

After applying the first filter, the WGS data were downloaded from Franklin, and the remaining filters were applied manually. Second, only variants consistent with homozygous autosomal recessive and X-linked inheritance in the corresponding individuals were retained. Third, the functional impact of variants was assessed by focusing on coding effects likely to disrupt protein function, including frameshift, nonsynonymous missense, nonsense, splice acceptor/donor site, start-gain, start-loss, stop-gain, stop-loss, and in-frame variants. Fourth, because DNA was extracted from purified sperm samples of men with infertility, variants were cross-referenced with the Human Protein Atlas and the Genotype-Tissue Expression (GTEx) Portal (https://www.gtexportal.org/home/). Genes showing testis-specific expression were selected for further investigation. Fifth, variants were further filtered based on their potential relevance to infertility-related pathways and conditions. Sixth, all retained variants were annotated according to the ACMG guidelines^31^. Finally, the expression of the detected genes was verified in sperm cells using HISTA (https://conradlab.shinyapps.io/HISTA/). This multitier filtering strategy enabled the identification of candidate variants with high confidence for further investigation.

### Laser microdissection of individually selected sperm

For laser microdissection, 3 µL of the sperm suspension from the remainder of the samples used for sequencing (Samples 1 and 5) was applied to a Membrane Slide NF 1.0 PEN (Zeiss) and allowed to dry on a 37 °C heating plate. The adhered sperm cells were fixed with methanol, followed by DAPI staining (Sigma-Aldrich) to visualize nuclear DNA. For each sample, 25 individual sperm cells were microdissected in quadruplicate using the PALM® MicroBeam Microdissection System (Carl Zeiss AG). DNA extraction was performed using the QIAamp DNA Micro Kit (Qiagen). After an extended 16-h proteinase K digestion to ensure complete lysis, the lysate was processed according to the manufacturer’s instructions, with glycogen (Thermo Fisher Scientific) added to improve DNA recovery. The final eluate was concentrated to a total volume of approximately 5–6 µL for subsequent Sanger-based confirmation of the selected variants.

### Validation of genetic variants using sanger sequencing

Sanger sequencing was performed to validate selected WGS-detected variants in *PROCA1*, *CHRNA5*, *NPIPB15*, and *RGPD3*, chosen based on the clinical significance of the identified variants and the availability of sufficient residual DNA after the initial clinical and WGS analyses. Some variants could not be further assessed because of insufficient remaining DNA, which represents a technical limitation of the study. For this purpose, initial PCR amplification was carried out using primers listed in Supplemental Table 2, with MyTaq™ HS Red Mix in a 20 µL reaction volume containing 2 µL DNA (~ 10 ng/µL). The PCR product was analyzed via 1.5% agarose gel electrophoresis to verify the expected band sizes. Following confirmation, DNA bands of the correct size were excised from the gel after staining with GelStar™ (Thermo Fisher Scientific) and visualized under the UV light. The excised gel fragments were purified using the NucleoSpin® Gel kit (Macherey–Nagel). DNA was eluted in 15 µL Elution Buffer and quantified using a NanoDrop spectrophotometer (Thermo Fisher Scientific). Sanger sequencing was performed at Eurofins Genomics (Eurofins Genomics) sequencing service. The sequences were aligned with BLAST to confirm their unique genomic locations, and all variants were manually double-checked using the chromatograms.

**Table 2:** Candidate variants identified in high-quality (HQ) and poor-quality (PQ) sperm pools.

Sample ID	Gene	Chr	Start Position	Stop Position	Ref	Alt	dbSNP	Transcript	Protein Change	cDNA Change	Exon	Zygosity	Region	Effect	Depth	gnomAD (Genome)	Classification
High-quality selected sperm (HQ; motile with normal morphology, n = 6)																	
HQ1	*NPIPB15*	16	74391650	74391650	A	G	rs2868591	NM_001306094.2	p.Tyr301Cys	c.902A > G	8	Hom	Exonic	NS	59	0.63216	VUS
HQ2	*ANKRD36C*	2	95960544	95960544	C	T	rs4362598	NM_001393982.1	p.Gly311Asp	c.932G > A	10	Hom	Exonic	NS	25	0.96806	VUS
	*CLEC18B*	16	74421404	74421404	A	G	rs3851723	NM_001385192.1	–	c.-127-7T > C	2	Hom	Splice region	Other	34	0.73566	VUS
HQ3	*–*	–	–	–	–	–	–	–	–	–	–	–	–	–	–	–	–
HQ4	*ANKRD36C*	2	95960544	95960544	C	T	rs4362598	NM_001393982.1	p.Gly311Asp	c.932G > A	10	Hom	Exonic	NS	36	0.96806	VUS
	*NPIPB15*	16	74391650	74391650	A	G	rs2868591	NM_001306094.2	p.Tyr301Cys	c.902A > G	8	Hom	Exonic	NS	28	0.63216	VUS
	*OR10G2*	14	21634436	21634436	C	T	rs10138694	NM_001005466.2	p.Arg136His	c.407G > A	1	Hom	Exonic	NS	29	0.48351	VUS
HQ5	*PROCA1*	17	28703797	28703797	C	T	rs143950518	NM_001366301.1	p.Ala286Thr	c.856G > A	5	Hom	Exonic	NS	31	0.00095	VUS
	*KIAA0100*	17	28634534	28634534	T	C	rs148988447	NM_014680.5	p.Asn1018Ser	c.3053A > G	16	Hom	Exonic	NS	32	0.00116	VUS
	*CHRNA5*	15	78590583	78590583	G	A	rs16969968	NM_000745.4	p.Asp398Asn	c.1192G > A	5	Hom	Exonic	NS	30	0.57736	VUS
	*NPIPB15*	16	74391650	74391650	A	G	rs2868591	NM_001306094.2	p.Tyr301Cys	c.902A > G	8	Hom	Exonic	NS	22	0.36531	VUS
HQ6	*NPIPB15*	16	74391650	74391650	A	G	rs2868591	NM_001306094.2	p.Tyr301Cys	c.902A > G	8	Hom	Exonic	NS	39	0.63216	VUS
Poor-quality selected sperm(PQ; immotile with abnormal morphology, n = 6) sperm																	
PQ1	*NPIPB15*	16	74391460	74391460	G	A	rs6564065	NM_001306094.2	p.Ala238Thr	c.712G > A	8	Hom	Exonic	NS	33	0.547441	VUS
	*NPIPB15*	16	74391650	74391650	A	G	rs2868591	NM_001306094.2	p.Tyr301Cys	c.902A > G	8	Hom	Exonic	NS	34	0.63216	VUS
PQ2	*RGPD3*	2	106457045	106457045	C	T	rs62152530	NM_001144013.2	p.Asp111Asn	c.331G > A	4	Hom	Exonic	NS	33	0.49788	VUS
	*ANKRD36C*	2	95960544	95960544	C	T	rs4362598	NM_001393982.1	p.Gly311Asp	c.932G > A	10	Hom	Exonic	NS	27	0.96806	VUS
PQ3	*–*	–	–	–	–	–	–	–	–	–	–	–	–	–	–	–	–
PQ4	*NPIPB15*	16	74391650	74391650	A	G	rs2868591	NM_001306094.2	p.Tyr301Cys	c.902A > G	8	Hom	Exonic	NS	22	0.63216	VUS
	*NPIPB15*	16	74391671	74391671	C	T	rs11641596	NM_001306094.2	p.Pro308Leu	c.923C > T	8	Hom	Exonic	NS	25	0.43711	VUS
PQ5	*KIAA0100*	17	28634534	28634534	T	C	rs148988447	NM_014680.5	p.Asn1018Ser	c.3053A > G	16	Hom	Exonic	NS	28	0.00116	VUS
	*PROCA1*	17	28703797	28703797	C	T	rs143950518	NM_001366301.1	p.Ala286Thr	c.856G > A	5	Hom	Exonic	NS	27	0.00096	VUS
	*NPIPB15*	16	74391650	74391650	A	G	rs2868591	NM_001306094.2	p.Tyr301Cys	c.902A > G	8	Hom	Exonic	NS	28	0.63217	VUS
PQ6	*FOXO6*	1	41382200	41382200	G	–	–	NM_001291281.3	p.Arg404Glyfs*106	–	–	–	–	Frameshift	22	0.99734	Possibly Pathogenic
	*NPIPB15*	16	74391650	74391650	A	G	rs2868591	NM_001306094.2	p.Tyr301Cys	c.902A > G	8	Hom	Exonic	–	63	0.63216	VUS

Chr, chromosome; Ref, reference allele; Alt, alternate allele; dbSNP, Database of Single Nucleotide Polymorphisms; cDNA, complementary DNA; AA, amino acid; Hom, homozygous; NS, non-synonymous; VUS, variant of uncertain significance; gnomAD, Genome Aggregation Database; Genoox, genomic interpretation platform.

*FOXO6* (PQ6)—c.1008_1209 + 1ins, p.Arg404Glyfs*106. This variant represents a homozygous frameshift insertion at the exon–intron boundary, introducing a long GC-rich nucleotide stretch (5′-GGGACGCCCGCCTACTTCGGCGGCTGCAAGGGCGGCGCCTACGGCGGGGGGGGGGCTTCGGGCCGCCGGCGATGGGCGCTCTGCGCCGTCTGCCCATGCAGACCATCCAGGAGAACAAGCAGGCCAGCTTCGTGCCGGCCGCGGCGCCCTTCCGCCCTGGGGCGCTGCCCGCGCTGCTGCCGCCGCCGCCGCCCGCGCC-3′), which is predicted to cause premature termination of the protein (p.Arg404Glyfs*106).

### Statistical analysis

All statistical tests were conducted to descriptively compare variant count categories between HQ and PQ pools. For each variant class, the mean and standard deviation were calculated. Before comparison, data were tested for normality and homogeneity of variances. Since equal variances were not assumed, the Welch’s t-test (robust to unequal variances) was used to compare group means.

In addition to significance testing, effect size was assessed by calculating Cohen’s d, measuring the strength and direction of differences between the HQ and PQ groups. Cohen’s d was calculated using pooled standard deviations and adjusted for any unequal group sizes. A negative Cohen’s d indicated fewer variants in HQ than in PQ. Because HQ and PQ samples were derived from the same individuals and variant-count comparisons were exploratory, effect sizes and p-values were interpreted descriptively rather than as evidence of biologically distinct inherited variant burden between groups. The significance level was set at α = 0.05 for all tests. All analyses were conducted in R (RStudio version 2024.04.1) using standard statistical packages.

## Results

### Clinical and reproductive profiles of included subjects

The cohort comprised six male partners aged 21–30 years (mean ± SD: 25.5 ± 3.73), as shown in Supplemental Table 2. The mean sperm count was 37.33 ± 18.82 million/mL, with progressive motility averaging 12.17 ± 7.44% and non-progressive motility 31.33 ± 14.45%. Immotile sperm accounted for a higher proportion overall (56.5 ± 17.24%). Normal sperm morphology averaged 6.83 ± 3.31%.

Embryo quality varied, with grades ranging from G1 to G3. In this small cohort, higher-quality embryos were observed in cases with better reproductive outcomes. Positive β-hCG results, indicative of pregnancy, were observed in three cases, two of which resulted in live births (all female infants). Negative β-hCG outcomes were associated with lower embryo quality and/or failure of pregnancy progression.

Hormonal profiles of the female partners showed mean FSH and LH levels of 6.0 ± 2.75 mIU/mL and 8.82 ± 2.35 mIU/mL, respectively. In this small cohort, positive β-hCG outcomes were observed in cases with relatively lower gonadotropin levels, although no formal association analysis was performed.

### Alignment quality across high- and poor-quality sperm

To assess whether sequencing quality systematically differed between selected sperm of various phenotypic classes, we compared group-level quality control parameters between high-quality (HQ; motile, normal morphology, n = 6) and poor-quality (PQ; immotile, abnormal morphology, n = 6) sets of selected sperm (Supplemental Table 3). On average, HQ samples yielded 708.5 million reads (range 586.3–975.4 million) as compared to 729.0 million (range 586.3–867.1 million) for PQ samples, reflecting relatively small differences in output of sequence reads. These small output differences did not correspond to substantial differences in sequencing and alignment efficiencies, as both sets achieved equally high rates of mapping (HQ averaging 99.81%, range 99.74–99.85%; PQ averaging 99.73%, range 99.66–99.82%), and mean mapping quality scores were essentially identical between HQ (31.80 ± 0.14) and PQ (31.75 ± 0.10) sets, reflecting uniform base-level correspondence across the dataset.

The deepest coverage exhibited broader variation: HQ samples averaged 31.9× (range 25.4–44.7×), due to HQ1’s extremely high coverage, whereas PQ samples averaged 32.0× (range 25.4–38.3×). Median coverage estimates were in tight synchrony (HQ median = 29.0×; PQ median = 31.0×), reflecting the overall similarity between sets. Overall, these set-level comparisons indicate broadly similar sequencing output, mapping rates, and coverage depths between HQ and PQ sperm pools, reducing the likelihood that downstream observations were driven primarily by systematic differences in sequencing or alignment performance.

### Descriptive comparison of variant counts between HQ and PQ sperm pools

A total of 2301, 2249, 2118, 2387, 1935, and 2339 variants were identified in samples PQ1, PQ2, PQ3, PQ4, PQ5, and PQ6, respectively, and were included for downstream annotation in the Franklin platform. Similarly, for the HQ cohort, 2514, 1978, 1951, 2361, 2118, and 1599 variants in HQ1, HQ2, HQ3, HQ4, HQ5, and HQ6 were selected for subsequent downstream annotation within the same platform. These variants were further subjected to stringent filtering criteria following comprehensive annotation to evaluate their potential functional and clinical relevance (Supplemental Table 3). We descriptively compared variant counts between HQ and PQ sperm pools (Supplemental Table 3/Supplemental Table 4). Across several variant categories, PQ sperm pools showed numerically higher average counts than HQ pools. For single-nucleotide variants (SNVs), HQ samples averaged 3.29 million (± 0.52 million), while PQ samples averaged 3.54 million (± 0.14 million). PQ samples also showed more structural changes, including deletions (PQ: 385,656 ± 39,050 vs. HQ: 348,967 ± 83,675) and insertions (PQ: 382,340 ± 41,943 vs. HQ: 338,408 ± 83,097). Indel counts followed the same trend (PQ: 3285 ± 468 vs. HQ: 2901 ± 957), as did sequence alterations (PQ: 52,133 ± 7922 vs. HQ: 45,977 ± 16,628). Despite these consistent trends, Welch’s t-tests did not find significant differences between groups for any category (all p > 0.28), likely due to limited power from the small sample size. However, effect size estimates (Cohen’s d ≈ − 0.5 to − 0.7 across categories) showed a consistent numerical direction, with lower counts in HQ than in PQ pools. None of these comparisons reached statistical significance (all *p* > 0.28). Because HQ and PQ sperm pools originated from the same individuals, these numerical differences were treated as descriptive observations rather than evidence of biologically distinct inherited variant burden.

### Descriptive comparison of splice-site and protein-altering variant annotations

Next, we looked at variant types expected to have the strongest effects on proteins and transcript integrity (Supplemental Table 5). Supplementary Table 5 summarizes the most severe VEP consequences across all variant classes. In many of these categories, as shown in Table 1, PQ sperm pools showed numerically higher counts than HQ pools. Frameshift variants, which disrupt the reading frame and can create premature stop codons, were more common in PQ (290 ± 32 vs. 269 ± 61; Cohen’s d = − 0.45). Stop-gained mutations (which introduce premature termination) were also more frequent in PQ (132 ± 15 vs. 123 ± 24; d = − 0.46), as were start-lost variants (36 ± 5 vs. 32 ± 10; d = − 0.54) and stop-lost variants (62 ± 4 vs. 59 ± 7; d = − 0.54).

Higher counts were observed in PQ pools across several splice-related annotation categories (Table 1). Canonical splice acceptor and donor variants were elevated (PQ: 244 ± 14 vs. HQ: 226 ± 42, d = − 0.57; PQ: 339 ± 30 vs. HQ: 312 ± 50, d = − 0.63). Additionally, PQ sperm had more splice donor region variants (688 ± 74 vs. 626 ± 122; d = − 0.62), splice donor 5th-base variants (255 ± 21 vs. 235 ± 46; d = − 0.56), and splice region variants (4186 ± 373 vs. 3858 ± 737; d = − 0.56).

PQ pools also showed higher counts of splice polypyrimidine tract variants (3534 ± 272 vs. 3267 ± 575; d = − 0.59). These differences were descriptive and were not interpreted as evidence of true group-specific disruption of splicing. Beyond splicing, alterations at the coding level showed a similar pattern, as shown in Table 1. PQ pools showed more missense variants (10,063 ± 1,207 vs. 9260 ± 1993; d = − 0.49), along with more inframe insertions (122 ± 34 vs. 105 ± 45; d = − 0.42) and inframe deletions (156 ± 41 vs. 140 ± 56; d = − 0.33). Notably, PQ pools showed higher counts of protein-altering variants (4.2 ± 2.3 vs. 2.2 ± 1.7; d = − 0.98), a category that includes non-synonymous changes. Rare categories like incomplete terminal codon variants had the largest relative effect (d = − 1.38), although absolute counts were still low.

While none of these group differences reached statistical significance (all p > 0.12), the effect sizes were generally moderate to strong (Cohen’s d ≈ − 0.4 to − 1.4), with the largest differences found for protein-altering variants, splice-site-related variants, and stop/start codon variants. Although several protein-altering and splice-related annotation categories were observed at a higher frequency in PQ pools, none of these differences reached statistical significance. Given the shared germline background of the paired HQ and PQ sperm pools derived from the same individuals, these observations were not interpreted as evidence of genuine sequence-level differences between sperm quality groups.

### Descriptive comparison of coding variant annotations between HQ and PQ pools

We next compared the distribution of coding variant consequences between the HQ and PQ sperm pools (Supplementary Table 6). Because Supplementary Table 6 is restricted to coding consequence annotations, these classifications may differ as a result of transcript context and coding-specific filtering.

In several coding annotation categories, as shown in Table 1, PQ pools showed numerically higher counts than HQ pools. Among the most harmful categories, frameshift variants, which disrupt the reading frame and often create truncated proteins, were more frequent in PQ (166 ± 17 vs. 156 ± 30; Cohen’s d = − 0.42). PQ pools also contained more stop-gained (nonsense) mutations (75 ± 7 vs. 71 ± 12; d = − 0.42), incomplete terminal codon variants (105 ± 30 vs. 91 ± 42; d = − 0.38), and start-lost variants (12 ± 2 vs. 11 ± 5; d = − 0.39). Protein-altering variants also showed strong differences (2.3 ± 1.9 in PQ vs. 0.5 ± 0.8 in HQ; d = − 1.27), even though these were uncommon. In contrast, stop-lost and stop-retained variants occurred at similar rates in both groups (22 each; d ≈ 0.00 for stop-lost; − 0.15 for stop-retained).

Missense and synonymous variants, which make up the largest groups of coding variation, were also higher in PQ pools (Table 1). PQ pools had an average of 8522 ± 955 missense variants compared to 7862 ± 1614 in HQ (d = − 0.50), and 9231 ± 1246 synonymous variants compared to 8398 ± 1981 in HQ (d = − 0.50). Other, less frequent but directionally consistent differences were seen in in-frame insertions (138 ± 36 in PQ vs. 122 ± 49 in HQ; d = − 0.36) and in-frame deletions (0.7 ± 0.8 vs. 0.3 ± 0.5; d = − 0.49). Coding sequence variants, a broad category, also trended higher in PQ pools (5.0 ± 1.4 vs. 4.5 ± 2.8; d = − 0.22).

None of these comparisons reached statistical significance (all p > 0.41), likely due to the small sample size, but effect sizes suggested moderate to large differences between groups, particularly for protein-altering variants (d = − 1.27), missense, synonymous, and frameshift variants (d ≈ − 0.4 to − 0.5). Overall, no statistically significant differences were observed in coding variant annotation categories between HQ and PQ pools. These observations were therefore interpreted descriptively rather than as evidence of clear differences in coding variant burden between sperm quality groups.

### Descriptive comparison of SIFT and PolyPhen annotations

To further assess the expected impact of coding variants, we compared SIFT and PolyPhen predictions between HQ and PQ sperm pools (Supplemental Table 7). Using both prediction methods, as shown in Table 1, PQ pools showed slightly higher counts in several SIFT- and PolyPhen-annotated categories. According to SIFT, PQ pools had more harmful variants (1291 ± 137 vs. 1182 ± 223 in HQ; Cohen’s d = − 0.59) and harmful variants with low confidence (518 ± 64 vs. 487 ± 115; d = − 0.33). PQ pools also showed greater counts for tolerated variants (5116 ± 579 vs. 4714 ± 964; d = − 0.51) and low-confidence tolerated variants (1233 ± 175 vs. 1125 ± 267; d = − 0.48).

Similarly, PolyPhen predictions indicated that PQ pools had more likely damaging variants (534 ± 67 vs. 494 ± 92; d = − 0.50) and possibly damaging variants (621 ± 78 vs. 576 ± 126; d = − 0.43) (Table 1). PQ also had more benign variants (5995 ± 698 vs. 5497 ± 1148; d = − 0.52) and variants with uncertain effects (736 ± 87 vs. 689 ± 135; d = − 0.42).

Although no group differences reached statistical significance (all *p* > 0.33), effect sizes were moderate across categories (Cohen’s d ≈ − 0.4 to − 0.6), showing a consistent numerical direction across both harmful and benign prediction categories in PQ pools. Overall, these comparisons did not reveal statistically significant differences in SIFT- or PolyPhen-based annotation counts between HQ and PQ pools. These results were interpreted as descriptive annotation patterns rather than evidence of a biologically distinct coding variant burden, and the functional significance of these predicted effects remains to be experimentally validated.

### Overall variants detection

WGS analysis identified a total of 42 unique high-confidence variants affecting 32 genes across 12 sperm samples (HQ = 6, PQ = 6). Raw WGS data after applying the first filter were downloaded from Franklin and are included as Supplemental Table 8. The majority were SNVs located within exonic regions, with a smaller proportion affecting splice sites (Table 2). All detected variants were consistent with homozygous genotypes in the corresponding individuals, as inferred from pooled sperm DNA. Based on ACMG guidelines, most alterations were classified as variants of uncertain significance (VUS). The only exception was a frameshift insertion in *FOXO6* (c.1008_1209 + 1ins, p.Arg404Glyfs*106) identified in PQ6, which was classified as possibly pathogenic and represented the most notable exploratory finding in this dataset.

#### Sample-based variant distribution

Among poor-quality sperm, PQ1 harbored two variants in *NPIPB15* (p.Ala238Thr, c.712G > A; p.Tyr301Cys, c.902A > G), both variants were consistent with homozygous genotypes. PQ2 carried variants consistent with homozygous genotypes in *RGPD3* (p.Asp111Asn, c.331G > A) and *ANKRD36C* (p.Gly311Asp, c.932G > A). PQ3 was negative for reportable variants. PQ4 contained two *NPIPB15* variants: the recurrent p.Tyr301Cys (c.902A > G) and an additional p.Pro308Leu (c.923C > T), both variants were consistent with homozygous genotypes. PQ5 carried rare variants consistent with homozygous genotypes in *KIAA0100* (p.Asn1018Ser, c.3053A > G, gnomAD AF = 0.00116) and *PROCA1* (p.Ala286Thr, c.856G > A, gnomAD AF = 0.00095), together with the recurrent *NPIPB15* p.Tyr301Cys. The most notable exploratory finding was in PQ6, in which a *FOXO6* frameshift variant (p.Arg404Glyfs*106, c.1008_1209 + 1ins) was detected in addition to the recurrent *NPIPB15* p.Tyr301Cys.

In high-quality sperm, HQ1 contained the recurrent *NPIPB15* p.Tyr301Cys (c.902A > G). HQ2 harbored *ANKRD36C* p.Gly311Asp (c.932G > A) and a splice-site alteration in *CLEC18B* (c.-127-7T > C), both variants were consistent with homozygous genotypes. HQ3 did not reveal reportable variants. HQ4 carried three variants consistent with homozygous genotypes: *ANKRD36C* p.Gly311Asp (c.932G > A), *NPIPB15* p.Tyr301Cys (c.902A > G), and *OR10G2* p.Arg136His (c.407G > A). HQ5 harbored a combination of rare and common variants, including *KIAA0100* p.Asn1018Ser (c.3053A > G), *PROCA1* p.Ala286Thr (c.856G > A), *CHRNA5* p.Asp398Asn (c.1192G > A, gnomAD AF ~ 0.57), and again the recurrent *NPIPB15* p.Tyr301Cys. Finally, HQ6 demonstrated the recurrent *NPIPB15* p.Tyr301Cys. Detailed annotations for all variants, including Ref/Alt alleles, read depth, zygosity, and classification, are provided in Table 2.

In contrast to the other samples, HQ3 and PQ3 did not yield any reportable variants. Both samples, however, showed sequencing and alignment quality metrics within the range observed for the other analyzed samples, arguing against technical failure as the cause. HQ3 showed a mapping rate of 99.79%, an unmapped read fraction of 0.21%, a mean depth-related value of 15.39, a mean mapping quality of 31.70, and a general error rate of 0.92, while PQ3 showed corresponding values of 99.81%, 0.19%, 16.01, 31.75, and 0.89. Thus, after application of the stringent filtering criteria described in the Materials and Methods section, no exonic or splice-site variants in these two samples met the applied quality and population frequency thresholds.

#### Recurrent versus unique variants

Several variants were recurrent across samples, whereas others were detected only in individual cases. The most frequent was *NPIPB15* p.Tyr301Cys (c.902A > G, classified as a VUS and consistent with a homozygous genotype), detected in seven samples across both HQ and PQ groups (HQ1, HQ4, HQ5, HQ6, PQ1, PQ4, PQ5, PQ6; Table 2). This variant was consistently observed with relatively high allele frequency in gnomAD. Another recurrent finding was *ANKRD36C* p.Gly311Asp (c.932G > A, VUS), which occurred in HQ2, HQ4, and PQ2. In addition, *KIAA0100* p.Asn1018Ser (c.3053A > G, VUS) and *PROCA1* p.Ala286Thr (c.856G > A, VUS) were detected in both HQ5 and PQ5.

Several variants were detected only in individual HQ samples under the applied filtering criteria. These included *CLEC18B* (splice region c.-127-7T > C, VUS; HQ2), *OR10G2* p.Arg136His (c.407G > A, VUS; HQ4), and *CHRNA5* p.Asp398Asn (c.1192G > A, VUS; HQ5). All were classified as variants of uncertain significance and were restricted to single HQ samples.

Other variants were detected only in individual PQ samples under the applied filtering criteria. These included *NPIPB15* p.Ala238Thr (c.712G > A, VUS; PQ1), *NPIPB15* p.Pro308Leu (c.923C > T, VUS; PQ4), and *RGPD3* p.Asp111Asn (c.331G > A, VUS; PQ2). Notably, PQ6 showed a *FOXO6* frameshift variant (p.Arg404Glyfs*106, possibly pathogenic), which was the only predicted loss-of-function variant observed in the cohort. Overall, the dataset included recurrent missense variants, particularly *NPIPB15* p.Tyr301Cys and *ANKRD36C* p.Gly311Asp, together with additional variants detected only in individual HQ or PQ samples under the applied filtering criteria. The *FOXO6* frameshift in PQ6 represented the most disruptive candidate variant observed.

#### Zygosity and allele frequency patterns

All identified variants were consistent with homozygous genotypes in the individuals, inferred from pooled sperm DNA. Population allele frequencies from gnomAD showed considerable variation, ranging from very rare alleles such as *KIAA0100* p.Asn1018Ser (c.3053A > G, AF = 0.00116, VUS) and *PROCA1* p.Ala286Thr (c.856G > A, AF = 0.00095, VUS) to moderately common variants including *NPIPB15* p.Tyr301Cys (c.902A > G, AF = 0.36–0.63, VUS) and *OR10G2* p.Arg136His (c.407G > A, AF = 0.48, VUS), and very common variants such as *ANKRD36C* p.Gly311Asp (c.932G > A, AF = 0.96, VUS). The *FOXO6* frameshift insertion (c.1008_1209 + 1ins, not present in gnomAD) was unique to PQ6 and represented the strongest exploratory candidate variant with potential functional relevance (Table 2).

### Validation of WGS results by Sanger sequencing

**Fig. 2:**
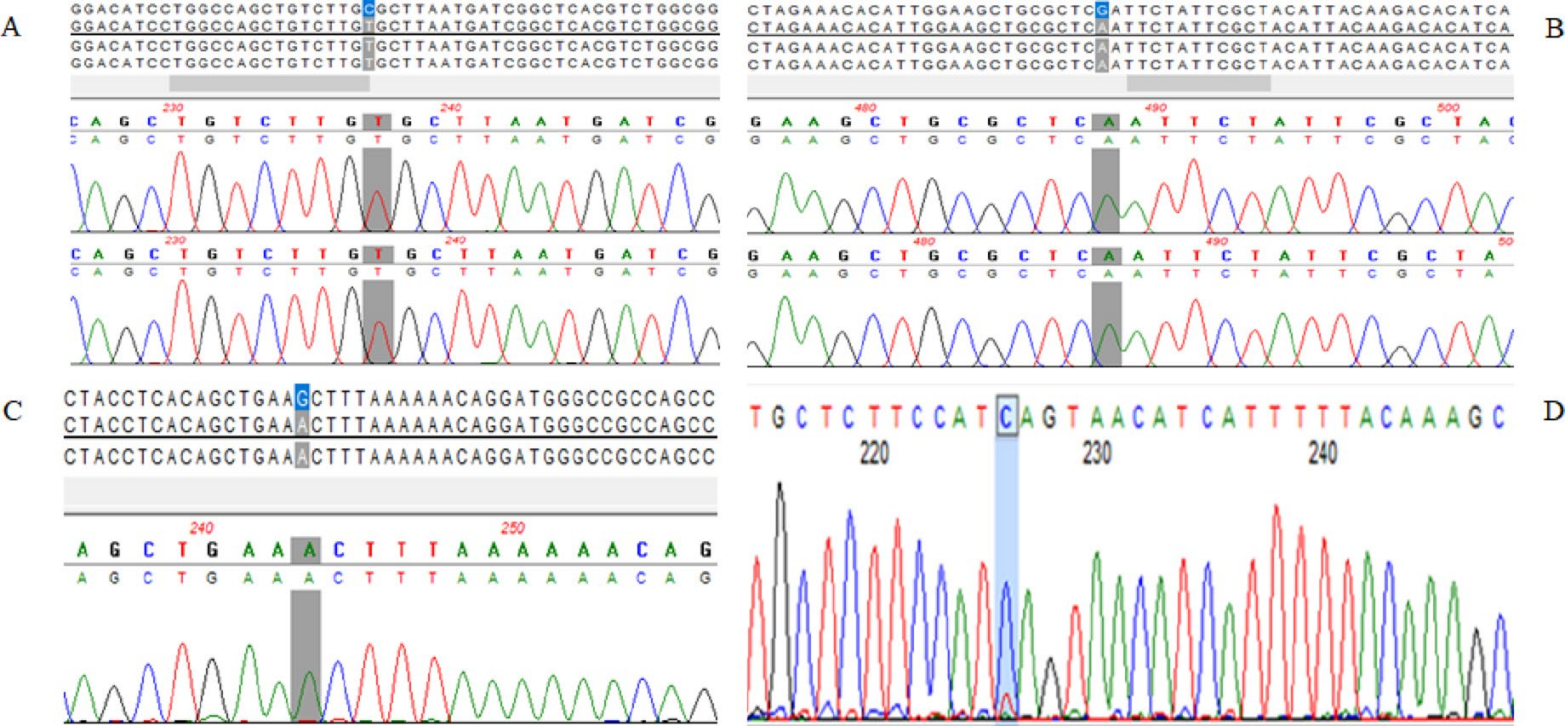
The Sanger sequencing chromatogram validated the WGS-detected variants in (**A**) represent two variants in *PROCA1*, similarly (**B**) represent two variants in *CHRNA5*, (**C**) represent *NPIPB15*, (**D**) represents one variant in *RGPD3* respectively*.* The detected mutations are highlighted with a grey/blue colour.

Sanger sequencing was performed to validate WGS-detected variants in *PROCA1*, *CHRNA5*, *NPIPB15*, and *RGPD3*. Sanger chromatograms confirmed the presence of the respective variants at the corresponding genomic positions in these four genes, as shown in Fig. 2, thereby supporting the accuracy of the WGS calls at the selected loci. These genes were selected for validation based on the clinical significance of the identified variants and the availability of sufficient residual DNA after WGS, derived either from isolated DNA or the original sample material. Additionally, in two samples for which purified sperm from the ICSI procedure remained available, microdissection was used to isolate 25 individual spermatozoa (Supplementary Figures 1A and 1B). While some selected variants were successfully validated, others could not be assessed due to insufficient residual DNA, thereby highlighting a technical limitation of the study.

## Discussion

In this study, we compared the genomic profiles of pooled DNA derived from individually selected HQ and PQ sperm collected from the male partners of subfertile couples. By selecting individual spermatozoa and pooling them according to morphology and motility immediately before ICSI, we generated paired HQ and PQ sperm pools from the same individuals, enabling direct comparison between motile, morphologically normal sperm (HQ) and immotile, morphologically abnormal sperm (PQ). This pooled-sperm approach reduced bulk-semen heterogeneity at the level of sperm selection; however, because sequencing was performed on pooled sperm DNA, the resulting WGS data reflect pooled germline signal from the corresponding individual rather than single-sperm genotypes. Across descriptive comparisons, poor-quality (PQ) sperm pools frequently showed numerically higher counts of SNVs, indels, structural variants, and coding variants compared with high-quality (HQ) sperm pools, although these differences did not reach statistical significance. The same pattern persisted when evaluating variants predicted to have functional or deleterious consequences. However, because HQ and PQ pools originated from the same individuals, these observations were interpreted descriptively rather than as evidence of biologically distinct inherited variant burden between sperm quality groups.

Collectively, these findings do not support a clear sequence-level difference in inherited SNV or coding variant burden between HQ and PQ sperm pools. Instead, the data are more consistent with descriptive numerical differences across pooled variant annotations, while the broader literature supports an association between male infertility and genomic instability at the level of chromosomal integrity, DNA fragmentation, and aneuploidy. This finding aligns with evidence that male infertility is frequently accompanied by subtle genomic abnormalities. Recent WGS studies of men with idiopathic sperm dysfunction have reported elevated burdens of SNVs, structural variants, and unique missense and frameshift mutations in key flagellar and spermatogenic genes^8,17,32^. These findings support the concept that deleterious variants affecting spermatogenesis or sperm structure may be relevant to abnormal sperm morphology and reduced motility. Consistent with this, several candidate variants identified in our cohort occurred in genes with plausible roles in male reproductive biology, although the descriptive differences observed between HQ and PQ pools were not interpreted as evidence of increased inherited mutational load in PQ sperm.

One explanatory framework for these observations is genome instability in male infertility. Subfertile men, particularly those with idiopathic oligoasthenoteratozoospermia, often exhibit evidence of genomic instability, including elevated sperm DNA fragmentation and increased chromosomal aneuploidy^33^. Defective spermatozoa commonly harbor unrecognized DNA integrity problems, and men with teratozoospermia have been shown to have significantly higher sperm DNA fragmentation and abnormal chromosomal segregation compared with normozoospermic men^34^. These prior observations are most directly relevant to chromosomal instability and DNA integrity rather than to inherited SNV burden. In our study, PQ pools showed numerically higher counts across several variant annotation categories, but none of these differences were statistically significant and, given the shared germline background of paired HQ and PQ pools, were interpreted descriptively. Accordingly, our data are best considered hypothesis-generating in relation to genome instability at the chromosomal and structural level, rather than as evidence that poor-quality sperm harbor more inherited coding mutations.

It is important to note that all sperm from an individual share the same germline genome; therefore, variants consistent with homozygous germline genotypes in the corresponding individual are expected to be present in both HQ and PQ fractions and cannot account for differences in sperm quality^35^. This highlights the importance of post-meiotic or de novo alterations as a possible contributor to sperm heterogeneity. Such mutations may emerge during spermatogenesis as a consequence of DNA fragmentation, oxidative stress, defective chromatin packaging, or impaired DNA repair^36–38^. These processes may contribute to mosaicism within the sperm population and could potentially contribute to observed heterogeneity across sperm subpopulations. Consequently, poor-quality sperm have been reported to show increased DNA fragmentation, chromosomal abnormalities, and mosaic variants^36–38^. Recent evidence further suggests that sperm subpopulations, particularly in subfertile or older men, may harbor pathogenic variants at unexpectedly high frequency, likely reflecting clonal expansions or instability in spermatogonial stem cells^39^. Together, these observations support a model in which acquired genomic instability, rather than constitutional mutations, drives much of the variation in sperm quality.

However, not all studies report dramatic genomic differences between good- and poor-quality sperm. Netherton et al. examined ejaculates from healthy men and compared DNA from morphologically “good” versus “poor” sperm within the same individual, finding global SNP profiles to be highly similar^40^. This observation is consistent with the expectation that many inherited sequence variants are shared across sperm from the same individual. In this context, the broadly similar constitutional variant background between paired HQ and PQ sperm pools is biologically plausible. At the same time, the descriptive numerical differences observed in our dataset may still reflect biologically relevant variation associated with sperm quality, particularly in relation to genomic integrity and structural instability. Although these findings should be interpreted with caution because of the small sample size, they support the view that poor-quality sperm may carry a greater burden of genomic abnormalities that merit investigation in larger cohorts. This apparent discrepancy can be partly explained by differences in study design. Netherton et al. evaluated intra-ejaculate variation within the same man^40^, whereas our study examined paired HQ and PQ sperm pools obtained from subfertile individuals and considered these findings in the broader clinical context of male infertility. Men predisposed to producing poor-quality sperm because of genetic, environmental, or testicular microenvironmental factors may generate sperm populations with increased genomic instability, even when constitutional sequence variation remains largely shared within the same ejaculate. Accordingly, our findings may indicate that the principal differences between HQ and PQ sperm are more likely to involve genome integrity and structural abnormalities than major shifts in inherited variant background. While the magnitude of these differences should be interpreted cautiously in light of the limited cohort size, the consistent direction of the observed patterns supports their relevance as an exploratory signal for further study.

In our targeted analysis, we identified a broad spectrum of variants across exonic and splice-related regions, the majority of which were classified as variants of uncertain significance (VUSs). Among these, *NPIPB15* p.Tyr301Cys was the most frequently detected recurrent variant, appearing in seven independent samples. Although *NPIPB15* has not previously been established as a male infertility gene, high expression of *NPIPB15* mRNA has been reported in spermatogenic cells^41^. However, given its high population allele frequency, *NPIPB15* p.Tyr301Cys is unlikely to represent a highly penetrant monogenic cause of male infertility. In addition, because *NPIPB15* belongs to a paralogous gene family, mapping ambiguity in short-read WGS data cannot be excluded and may have contributed to its apparent recurrence. Accordingly, this variant is interpreted cautiously as either a common benign polymorphism or a recurrent signal influenced by mapping limitations, rather than as direct evidence of a pathogenic driver of spermatogenic failure. Its recurrent detection across multiple samples makes it an exploratory candidate for further investigation; however, recurrence alone is insufficient to establish pathogenicity or a specific role in sperm dysfunction. In addition, recurrent variants were also observed in *ANKRD36C* and *RGPD3*. Previously, it was reported that mutant mice containing SNV and/or frameshift mutation in *ANKRD36C* demonstrated a mottled retina with photoreceptor degeneration and male infertility characterized by oligozoospermia and asthenozoospermia^17^. Given the high allele frequency in the general population, the *ANKRD36C* p.Gly311Asp is most consistent with the benign polymorphism and is unlikely to represent a high-penetrance causal factor for infertility. Taken together, these recurring variants across multiple samples and their biological plausibility suggest that *NPIPB15*, *ANKRD36C*, and *RGPD3* should be regarded as candidate genes warranting further investigation in larger human cohorts, rather than confirmed infertility-associated genes based on the present data alone.

Many of the recurrent variants identified in this study remain classified as VUS due to the absence of clear functional evidence. Although some variants, particularly those detected under the applied filtering criteria in individual PQ samples, were repeatedly identified across multiple samples, their contribution to male infertility remains elusive. Their recurrence and presence in testis-expressed genes suggest a possible role in spermatogenesis, but this cannot be confirmed without direct functional testing. As highlighted in recent studies, genetic association alone is insufficient to establish pathogenicity; in vitro or in vivo assays are needed to determine whether these variants, including those found in *NPIPB15*, *ANKRD36C*, or *RGPD3*, have a true biological impact^42^. Without such experimental evidence, any conclusions regarding their clinical relevance must be drawn with caution.

The variant found to be of foremost interest in this dataset was the unique *FOXO6* frameshift mutation c.1008_1209 + 1ins; p.Arg404Glyfs*106. However, this *FOXO6* mutation was detected only in the poor-quality sperm sample PQ6. FOXO transcription factors play critical roles in cell-cycle regulation, oxidative stress response, and apoptosis^43,44^, and disruptions to FOXO signaling have been associated with impaired germ-cell survival and testicular dysfunction^45^. The impairment of the *FOXO* gene has been linked to the survival of germ cells and the functionality of the testes via the PI3K/PTEN signaling pathway^46^. The variant identified in this study is predicted to produce a truncated protein. However, because it was detected in only a single PQ sample and no independent constitutional reference sample or orthogonal quantitative validation was available, it should be interpreted as an exploratory candidate finding rather than definitive evidence of a causal mutation underlying male infertility. Although causality cannot be established from this single observation, the predicted truncation and the known role of *FOXO6* in cell survival pathways make this variant a strong candidate for functional follow-up in the context of male infertility. Additional rare variants consistent with homozygous genotypes in the corresponding individuals were also identified, including *KIAA0100* p.Asn1018Ser and *PROCA1* p.Ala286Thr. Although their roles in male infertility are not yet well defined, their rarity makes them reasonable candidates for exploratory follow-up^47^. However, their presence in pooled sperm DNA should be interpreted as reflecting candidate germline variation in the corresponding individuals rather than as PQ-specific mutational events.

To further assess the biological relevance of the identified candidate variants, we performed exploratory Gene Ontology (GO) and KEGG pathway enrichment analyses using the ToppGene Suite^48,49^. Although no category remained significant after multiple-testing correction, potentially reflecting the relatively small and functionally heterogeneous gene set, several biologically relevant themes emerged. Gene Ontology-based functional enrichment studies in male infertility have highlighted biological processes related to spermatogenesis, oxidative stress, chromatin organization, metabolism, and sperm function. Consistent with these reports, our analysis identified oxidative stress- and mitochondrial-related processes involving *SIRPA*, *MT-ND5*, and *GLS2*; oxygen-related and metabolic-response pathways involving *FOXO6*, *PNPLA3*, and *CHRNA5*; and DNA/chromatin regulation, particularly through *CHD8*. In addition, *CYP3A5* was linked to steroid metabolism, while phenotype-based annotations highlighted *PRAMEF10*, *FCGBP*, and *RGPD3* in relation to male infertility, abnormal spermatogenesis, and altered male reproductive morphology^50^. Although these findings did not remain significant after correction for multiple testing, they support the biological plausibility of the detected candidate genes and suggest that pathways related to oxidative stress, chromatin regulation, metabolism, and reproductive function may contribute to impaired sperm quality.

Our final interpretation brings together the genomic findings, clinical characteristics, and IVF outcomes. In the six male partners included in the study, there was a clear reduction in candidate variants detected only under the applied filtering criteria in PQ pools in the men who achieved live births (IDs 1, 3, and 5), who had few such variants and no high-impact mutations. These men also showed better morphology percentages (4–12%) and were able to produce quality embryos (G1–G2), suggesting that lower genomic instability may support improved fertilization and embryo development. In contrast, the men who did not have live births (IDs 2, 4, and 6) showed higher levels of candidate variants detected only in PQ pools under the applied filtering criteria, secondary mutations in *NPIPB15*, and, in the case of ID6, the only high-impact *FOXO6* frameshift mutation. These men also had poor progressive motility (0–20%) and a high proportion of immotile sperm (50–80%). Given the pooled-sperm design, small cohort size, and lack of direct validation of PQ-specific mutational processes, these observations should be regarded as preliminary associations rather than evidence that PQ-specific mutational load predicts reproductive outcome. The hormone levels of the female partners were within acceptable ranges in all couples studied, but the present dataset is too small to disentangle male and female contributions to IVF outcome with confidence. However, these associations are based on a very small cohort and should be interpreted as preliminary, requiring confirmation in larger, independent studies.

Although WGS currently remains best suited as an exploratory and research-oriented tool, its future clinical implementation may be most realistic in selected cases of unexplained male infertility, severe teratozoospermia, recurrent IVF/ICSI failure, or suspected genetic sperm defects. In routine clinical settings, more cost-effective stepwise approaches such as targeted sequencing panels, exome sequencing, chromosomal testing, Y-chromosome microdeletion analysis, DNA fragmentation assays, and complementary functional sperm assays may provide more immediate diagnostic value and may serve as practical entry points before broader genomic profiling is considered.

## Conclusion

Our findings suggest that poor-quality sperm pools may show numerically higher variant counts and features of possible genomic instability, although these observations were descriptive and not statistically significant. Because HQ and PQ sperm pools were derived from the same individuals, the data do not indicate clear differences in inherited constitutional variant burden between sperm quality groups. Instead, the results suggest a possible link between sperm quality and genomic integrity, particularly structural instability, which requires further investigation. We also identified recurrent candidate variants in *NPIPB15*, *ANKRD36C*, and *RGPD3*, along with a *FOXO6* frameshift variant in a single PQ sample. These findings remain exploratory, as functional validation and independent constitutional reference data were not available to establish pathogenicity or causality. Nevertheless, the recurrent detection of variants in testis-expressed genes highlights potentially relevant candidate loci for future studies of male subfertility.

Several limitations should be acknowledged. The small cohort size reduced statistical power and limited the generalizability of the findings. In addition, many detected variants remained classified as VUS, and their biological relevance could not be determined without direct functional testing. Reliance on population databases such as gnomAD may also incompletely capture regional or ethnic variation, potentially affecting variant frequency interpretation. Despite these limitations, the study supports the value of broad genomic assessment of morphology-selected sperm pools as an exploratory approach to identify candidate variants and patterns potentially associated with impaired sperm quality. Larger studies incorporating paired analyses of sperm subpopulations, functional validation, orthogonal confirmation, and longitudinal reproductive data will be needed to refine risk estimates and assess the potential clinical utility of sperm genomic profiling in male infertility.

## Supplementary Information

Below is the link to the electronic supplementary material.

Supplementary Material 1

## Data Availability

The whole genome sequencing (WGS) data in FASTQ format generated during this study have been deposited in the European Nucleotide Archive (ENA) under accession number PRJEB104315.
