# Supplementary material for: Genetic alterations in poor-quality individually selected sperm highlight candidate biomarkers for male subfertility
Source: Sci Rep. 2026 Apr 28;16:13643. doi: 10.1038/s41598-026-50620-0 (PMC13125504; doi:10.1038/s41598-026-50620-0)
Supplement: Supplementary file 1 — Supplementary Material 1 [file 41598_2026_50620_MOESM1_ESM.pdf]

# Genetic Alterations in Poor-Quality Individually Selected Sperm Highlight Candidate Biomarkers for Male Subfertility

Mohammad A. Al Smadi, Ph.D., [1*], Aftab Ali Shah, Prof. Dr., [2*], Muhammad Riaz Khan, Ph.D., [2],

Eman Alshdaifat, M.D.,[3], Ulrike Fischer, Prof. Dr., [4], Hashim Abdul-Khaliq, Prof. Dr., [4],

Eckart Meese, Prof. Dr., [4], Masood Abu-Halima, Ph.D., [4, 5]

**Affiliations**

[1] Department of Medical Laboratory Science, Al Al-Bayt University, Mafraq, Jordan.

[2] Department of Biotechnology, Faculty of Biological Sciences, University of Malakand, Chakdara, Khyber Pakhtunkhwa, Pakistan.

[3] Department of Obstetrics and Gynecology, Faculty of Medicine, Yarmouk University, Irbid, Jordan.

[4] Institute of Human Genetics, Saarland University, Homburg, Germany.

[5] Department of Paediatric Cardiology, Saarland University Hospital, Homburg, Germany.

[*] Contributed equally to this work

**Corresponding Author**

Masood Abu-Halima

Saarland University

Institute of Human Genetics

66421 Homburg, Germany

Tel: +49 (0) 6841 16 26289

Fax: +49 (0) 6841 16 26185

Email: Masood.Abu-Halima@uks.eu

**Supplemental Table 1.** Clinical information for participants included in sequencing analysis.

| Male Partner Sperm Parameters | | | | | | | IVF Outcome Parameters | Female Partner Hormonal Parameters | | | |
|---|---|---|---|---|---|---|---|---|---|---|---|
| **Male ID** | **Age (Year)** | **Sperm Count (million/ml)** | **Progressive Motility (Type A+B) %** | **Non-Progressive Motility (Type C) %** | **Immotile Sperm (Type D) %** | **Normal Morphology (%)** | **Embryo Quality Grade** | **β-hCG** | **Live Birth Outcome** | **FSH (mIU/ml)** | **LH (mIU/ml)** |
| **1** | 30 | 14 | 10 | 25 | 65 | 4 | G2 | + | Boy/Girl | 2.8 | 6.8 |
| **2** | 25 | 18 | 0 | 20 | 80 | 3 | G3 | — | No Live Birth | 8.6 | 11.4 |
| **3** | 30 | 40 | 11 | 60 | 29 | 12 | G1 | + | Girl | 3.3 | 8.7 |
| **4** | 24 | 42 | 20 | 28 | 52 | 6 | G2 | — | No Live Birth | 8.9 | 8.6 |
| **5** | 23 | 45 | 12 | 25 | 63 | 9 | G1 | + | Girl | 4.6 | 5.8 |
| **6** | 21 | 65 | 20 | 30 | 50 | 7 | G1/G2 | — | No Live Birth | 7.8 | 11.6 |
| **Mean (± SD)** | **25.5 (± 3.73)** | **37.33 (± 18.82)** | **12.17 (± 7.44)** | **31.33 (± 14.45)** | **56.5 (± 17.24)** | **6.83 (± 3.31)** | | | | **6.0 (± 2.75)** | **8.82 (± 2.35)** |

- Samples: n = 6 male partners of couples undergoing infertility treatment.
- Data are presented as mean ± standard deviation (SD) for each parameter.
- Abbreviations: β-hCG (+), positive; β-hCG (—), negative; FSH, Follicle-Stimulating Hormone; LH, Luteinizing Hormone; G1, Grade 1; G2, Grade 2; G3, Grade 3.

**Supplemental Table 2.** Primer sequences, expected product sizes, and sample distribution (Human, hg38, genomic DNA)

| Gene | Primer Name | Sequence (5′→3′) | Product Size (bp) | Sample IDs (HQ) | Sample IDs (PQ) |
|---|---|---|---|---|---|
| **FOXO6** | FOXO6_F | CAAGTTCCTGCGCATCAAGG | 849 | – | **PQ6** |
| | FOXO6_R | GGGCCGAATCGAAGTTGAAG | | | |
| **PROCA1** | PROCA1_F | TATTCTGCACCCTGACCACC | 529 | **HQ5** | **PQ5** |
| | PROCA1_R | CTGACCTAGGCACCAGCATG | | | |
| **KIAA0100** | KIAA0100_F | GAGCAGGTGGTTAAGGGCAT | 578 | **HQ5** | **PQ5** |
| | KIAA0100_R | AAGCAGCATGGGGAGTTGTT | | | |
| **CLEC18B** | CLEC18B_F | TCCACCTACCTCCTCACCAG | 527 | **HQ2** | – |
| | CLEC18B_R | AATGTCCCCAGGCCAATCAG | | | |
| **NPIPB15 (74391650)** | NPIPB15_F | AAGGTGCAGACTGTGTTGGT | 589 | **HQ1, HQ4, HQ5, HQ6** | **PQ1, PQ4, PQ5, PQ6** |
| | NPIPB15_R | GCGATTGTTCCACCTCATCCG | | | |
| **NPIPB15 (74391460)** | NPIPB15_F | TCTTTACTCCCCAGCTGTCG | 604 | **HQ1, HQ4, HQ5, HQ6** | **PQ1, PQ4, PQ5, PQ6** |
| | NPIPB15_R | CGGCCTCCTGAGTAGCTAAG | | | |
| **CHRNA5** | CHRNA5_F | AGGAAACAGAACCGACAGCT | 700 | **HQ5** | – |
| | CHRNA5_R | TGTCAACAATTCTGGCCCTC | | | |

- Primer pairs used for genomic PCR amplification of human loci (GRCh38/hg38), together with the corresponding sample IDs in which each primer pair was applied.
- Primer sequences are presented in 5′→3′ orientation.
- Product sizes indicate the expected genomic amplicon lengths.

- All primer pairs were evaluated for melting temperature (Tm), GC content, 3′ clamp, and potential primer–dimer formation using UCSC In-Silico PCR and NCBI Primer-BLAST.
- Sample IDs HQ1–HQ6 refer to high-quality sperm pools, whereas PQ1–PQ6 refer to poor-quality sperm pools (immotile, abnormal morphology; n = 6).

**Supplemental Table 3. Summary of sequencing quality, alignment, variant calling, and filtering metrics for the HQ and PQ groups**

| Workflow step | Tool(s) | Key metric | Group HQ | Group PQ |
|---|---|---|---|---|
| **Input sequencing data** | FASTQ | Total reads per sample | 586.3–975.4 million (mean 708.2 million) | 586.3–867.1 million (mean 729.4 million) |
| | | Mapped reads (%) | 99.74–99.85% (mean 99.81%) | 99.66–99.82% (mean 99.73%) |
| | | Mean mapping quality | 31.63–32.00 (mean 31.80) | 31.63–31.92 (mean 31.75) |
| | | Estimated depth of coverage (×) | 25.36–44.70× (mean 31.28×) | 25.36–38.29× (mean 31.99×) |
| **Raw read QC** | FastQC | Mean read length | 140.2–150.0 bp (mean 143.8 bp) | 140.2–144.0 bp (mean 142.0 bp) |
| **Trimming** | Cutadapt | Clipped reads | 2.88–7.05% (mean 4.53%) | 3.17–4.42% (mean 3.77%) |
| **Alignment** | BWA-MEM | Mapping rate | 99.74–99.85% (mean 99.81%) | 99.66–99.82% (mean 99.73%) |
| **BAM processing** | SAMtools + Picard | Duplicate rate | 17.64–24.10% (mean 20.87%) | 17.64–30.05% (mean 23.34%) |
| | | Mean mapping quality | 31.63–32.00 (mean 31.80) | 31.63–31.92 (mean 31.75) |
| | | General error rate | 0.67–1.00% (mean 0.88%) | 0.69–0.91% (mean 0.80%) |
| **Variant calling** | GATK HaplotypeCaller | Raw variants per sample | 2.76–4.72 million (mean 4.03 million) | 4.02–4.65 million (mean 4.36 million) |
| **Raw variant composition** | GATK/VEP annotation | SNVs | 3.29 million (81.96%) | 3.54 million (81.16%) |
| | | Deletions | 0.349 million (8.57%) | 0.386 million (8.82%) |
| | | Insertions | 0.338 million (8.29%) | 0.382 million (8.75%) |
| **Filtering steps** | Franklin | Variants retained for downstream annotation | 1,935–2,387 (mean 2,221) | 1,599–2,514 (mean 2,087) |
| **Final candidate variants** | Downstream prioritization | Final selected variants | 33 unique variants affecting 25 genes | 28 unique variants affecting 20 genes |

- HQ refer to high-quality sperm pools, whereas PQ refer to poor-quality sperm pools (immotile, abnormal morphology; n = 6).

**Supplemental Table 4.** Descriptive comparison of variant counts in high-quality (HQ) and poor-quality (PQ) sperm pools

| Metric | HQ Mean | HQ SD | PQ Mean | PQ SD | Cohen's d |
|---|---|---|---|---|---|
| **SNV** | 3293840 | 515054 | 3537305 | 141139 | -0,645 |
| **Deletion** | 348967 | 83675 | 385656 | 39050 | -0,562 |
| **Indel** | 2901 | 957 | 3285 | 468 | -0,510 |
| **Insertion** | 338408 | 83097 | 382340 | 41943 | -0,667 |
| **Sequence alteration** | 45977 | 16628 | 52133 | 7922 | -0,473 |

- Samples: high-quality (HQ; motile with normal morphology, n = 6) and poor-quality (PQ; immotile with abnormal morphology, n = 6) sperm pools.
- Abbreviation: SNV, single-nucleotide variant.
- Data are presented as mean ± standard deviation (SD) for each variant category.
- Comparisons between groups were performed using Welch's t-test; none reached statistical significance (all $p > 0.28$).
- Effect sizes, expressed as Cohen's d, are provided as descriptive measures of difference between HQ and PQ pools. Negative values indicate lower counts in HQ pools than in PQ pools.

**Supplementary Table 5.** Genetic variant consequences in high-quality (HQ) and poor-quality (PQ) sperm pools based on the VEP "most severe" annotation.

| Consequence | HQ Mean | HQ SD | PQ Mean | PQ SD | Cohen's d |
|---|---|---|---|---|---|
| **3′ UTR variant** | 44682.17 | 8158.86 | 48341.50 | 3369.72 | -0.59 |
| **5′ UTR variant** | 7078.00 | 2360.76 | 7963.00 | 1768.19 | -0.42 |
| **TFBS ablation** | 40.67 | 7.76 | 43.83 | 8.98 | -0.38 |
| **TF binding site variant** | 11588.17 | 1579.64 | 12268.17 | 513.42 | -0.58 |
| **Coding sequence variant** | 1.67 | 0.82 | 1.83 | 0.75 | -0.21 |
| **Downstream gene variant** | 150428.17 | 27317.20 | 163109.50 | 10675.04 | -0.61 |
| **Frameshift variant** | 268.50 | 60.90 | 290.17 | 31.96 | -0.45 |
| **Incomplete terminal codon variant** | 0.67 | 0.82 | 2.00 | 1.10 | -1.38 |
| **Inframe deletion** | 139.50 | 55.87 | 155.67 | 41.09 | -0.33 |
| **Inframe insertion** | 105.00 | 45.30 | 122.00 | 34.24 | -0.42 |
| **Intergenic variant** | 1241592.33 | 204686.68 | 1339087.67 | 57324.73 | -0.65 |
| **Intron variant** | 2149585.33 | 378261.71 | 2330287.33 | 125979.74 | -0.64 |
| **Mature miRNA variant** | 34.33 | 9.16 | 37.00 | 5.44 | -0.35 |
| **Missense variant** | 9259.67 | 1992.83 | 10063.17 | 1206.80 | -0.49 |
| **Non-coding transcript exon variant** | 83922.17 | 14170.58 | 90408.00 | 5802.27 | -0.60 |
| **Non-coding transcript variant** | 0.17 | 0.41 | 0.50 | 0.55 | -0.69 |
| **Protein-altering variant** | 2.17 | 1.72 | 4.17 | 2.32 | -0.98 |
| **Regulatory region variant** | 133575.50 | 21016.14 | 143549.00 | 6600.15 | -0.64 |
| **Splice acceptor variant** | 226.17 | 41.68 | 243.83 | 13.54 | -0.57 |
| **Splice donor 5th-base variant** | 234.67 | 46.31 | 254.83 | 21.04 | -0.56 |
| **Splice donor region variant** | 625.50 | 121.99 | 688.00 | 73.59 | -0.62 |
| **Splice donor variant** | 312.33 | 50.20 | 338.67 | 30.37 | -0.63 |
| **Splice polypyrimidine tract variant** | 3267.33 | 574.62 | 3534.00 | 271.99 | -0.59 |
| **Splice region variant** | 3857.83 | 737.43 | 4186.33 | 373.20 | -0.56 |
| **Start lost** | 31.67 | 10.07 | 36.00 | 5.29 | -0.54 |
| **Stop gained** | 123.17 | 23.84 | 132.33 | 14.92 | -0.46 |
| **Stop lost** | 59.00 | 6.66 | 62.00 | 4.15 | -0.54 |
| **Stop retained variant** | 17.50 | 3.67 | 18.17 | 2.23 | -0.22 |
| **Synonymous variant** | 8608.33 | 2079.03 | 9474.83 | 1334.00 | -0.50 |
| **Upstream gene variant** | 180422.17 | 33830.45 | 196012.33 | 15430.57 | -0.59 |

- Counts are summarized according to the VEP Sequence Ontology "most severe" consequence, that is, the single highest-impact consequence assigned per variant across all overlapping transcripts and genomic contexts.
- Samples: high-quality (HQ; motile with normal morphology, n = 6) and poor-quality (PQ; immotile with abnormal morphology, n = 6) sperm pools.
- Data are reported as mean ± standard deviation (SD) across samples in each group for each consequence category.
- Comparisons between groups were performed using Welch's t-test; none reached statistical significance (all $p > 0.12$).

- Effect sizes are given as Cohen's d as descriptive measures across categories; negative values indicate lower counts in HQ pools than in PQ pools.

**Supplementary Table 6.** Coding consequence annotations in high-quality (HQ) and poor-quality (PQ) sperm pools.

| Consequence | HQ Mean | HQ SD | PQ Mean | PQ SD | Cohen's d |
|---|---|---|---|---|---|
| **Coding sequence variant** | 4.50 | 2.81 | 5.00 | 1.41 | -0.22 |
| **Frameshift variant** | 155.83 | 30.39 | 166.17 | 17.19 | -0.42 |
| **In-frame deletion** | 0.33 | 0.52 | 0.67 | 0.82 | -0.49 |
| **In-frame insertion** | 122.00 | 49.25 | 137.50 | 35.73 | -0.36 |
| **Incomplete terminal codon variant** | 90.83 | 41.94 | 104.83 | 29.79 | -0.38 |
| **Missense variant** | 7862.33 | 1614.03 | 8521.83 | 955.46 | -0.50 |
| **Protein-altering variant** | 0.50 | 0.84 | 2.33 | 1.86 | -1.27 |
| **Start lost** | 10.50 | 4.97 | 12.00 | 2.10 | -0.39 |
| **Start retained variant** | 1.83 | 0.98 | 1.67 | 0.82 | 0.18 |
| **Stop gained** | 71.33 | 11.72 | 75.33 | 6.83 | -0.42 |
| **Stop lost** | 22.33 | 2.42 | 22.33 | 2.73 | 0.00 |
| **Stop retained variant** | 10.50 | 1.87 | 10.83 | 2.56 | -0.15 |
| **Synonymous variant** | 8397.83 | 1980.78 | 9230.50 | 1245.56 | -0.50 |

- Counts are summarized for coding-related VEP consequence categories only.
- Samples: high-quality (HQ; motile with normal morphology, n = 6) and poor-quality (PQ; immotile with abnormal morphology, n = 6) sperm pools.
- Data are reported as mean ± standard deviation (SD) across samples in each group for each coding consequence category.
- Comparisons between groups were performed using Welch's t-test; none reached statistical significance (all $p > 0.41$).
- Effect sizes are given as Cohen's d as descriptive measures across categories; negative values indicate lower counts in HQ pools than in PQ pools.

**Supplemental Table 7.** Descriptive comparison of SIFT and PolyPhen impact predictions for coding variants in high-quality (HQ) and poor-quality (PQ) sperm pools

| Metric | HQ Mean | HQ SD | PQ Mean | PQ SD | Cohen's d |
|---|---|---|---|---|---|
| **Deleterious** | 1182 | 223.42 | 1291 | 137.16 | -0.59 |
| **Deleterious (low confidence)** | 487 | 115.44 | 518 | 63.92 | -0.33 |
| **Tolerated** | 4714 | 963.69 | 5116 | 579.23 | -0.51 |
| **Tolerated (low confidence)** | 1125 | 266.88 | 1233 | 174.99 | -0.48 |

| Metric | HQ Mean | HQ SD | PQ Mean | PQ SD | Cohen's d |
|---|---|---|---|---|---|
| **Benign** | 5497 | 1148.36 | 5995 | 698.14 | -0.52 |
| **Possibly damaging** | 576 | 125.71 | 621 | 78.11 | -0.43 |
| **Probably damaging** | 494 | 92.37 | 534 | 66.92 | -0.50 |
| **Unknown** | 689 | 135.33 | 736 | 87.46 | -0.42 |

- Samples: high-quality (HQ; motile, normal morphology; n = 6) and poor-quality (PQ; immotile, abnormal morphology; n = 6) sperm pools.
- Counts reflect coding variants with SIFT and PolyPhen impact predictions.
- Data are reported as mean ± standard deviation (SD) across samples in each group for each prediction category.
- Comparisons between groups were performed using Welch's t-test; none reached statistical significance (all $p > 0.33$).
- Effect sizes are given as Cohen's d as descriptive measures across categories; negative values indicate lower counts in HQ pools than in PQ pools.

**Supplemental Table 8:** Variant-level annotations for all detected candidate variants in high-quality (HQ) and poor-quality (PQ) sperm pools, including read depth, allele frequency, and pathogenicity classification.

| Gene | Variation Type | Chr | Start Position | Stop Position | Ref | Alt | dbSNP | Transcript | AA Change | Nucleotide | Exon | Zygosity | Region | Effect | Depth | gnomAD (Genome) | Genoox Classification | Source_file |
|---|---|---|---|---|---|---|---|---|---|---|---|---|---|---|---|---|---|---|
| PLIN4 | SNP | 19 | 4511338 | 4511338 | T | A | rs7259721 | NM_001367868.2 | p.Lys874Asn | c.2622A>T | 5 | Homozygous | Exonic | nonsynonymous | 41 | 0,71064556 | VUS | HQ1 |
| ANKRD36C | SNP | 2 | 95960544 | 95960544 | C | T | rs4362598 | NM_001393982.1 | p.Gly311Asp | c.932G>A | 10 | Homozygous | Exonic | nonsynonymous | 30 | 0,9680612 | VUS | HQ1 |
| FCGBP | SNP | 19 | 39877858 | 39877858 | A | C | rs12610683 | NM_003890.2 | p.Ser4284Ala | c.12850T>G | 24 | Homozygous | Exonic | nonsynonymous | 31 | 0,9993551 | VUS | HQ1 |
| NBPF8 | SNP | 1 | 120449388 | 120449388 | G | A | rs1553248447 | NM_001037501.5 | p.Arg325Gln | c.974G>A | 9 | Homozygous | Exonic | nonsynonymous | 64 | 0,6197496 | VUS | HQ1 |
| NPIPB15 | SNP | 16 | 74391650 | 74391650 | A | G | rs2868591 | NM_001306094.2 | p.Tyr301Cys | c.902A>G | 8 | Homozygous | Exonic | nonsynonymous | 59 | 0,6321651 | VUS | HQ1 |
| ZNF717 | SNP | 3 | 75737230 | 75737230 | T | C | rs3009024 | NM_001290208.3 | p.Lys798Arg | c.2393A>G | 5 | Homozygous | Exonic | nonsynonymous | 68 | 0,479239 | VUS | HQ1 |
| FCGBP | SNP | 19 | 39886260 | 39886260 | G | T | rs149109818 | NM_003890.2 | p.Ala3841Glu | c.11522C>A | 20 | Homozygous | Exonic | nonsynonymous | 21 | 0,5066646 | VUS | HQ1 |
| CROCCP2 | SNP | 1 | 16626498 | 16626498 | C | G | rs942268 | NR_026752.1 | | n.347G>C | 4 | Homozygous | Splice Region | Other | 53 | 0,75292456 | VUS | HQ2 |
| ANKRD36C | SNP | 2 | 95960544 | 95960544 | C | T | rs4362598 | NM_001393982.1 | p.Gly311Asp | c.932G>A | 10 | Homozygous | Exonic | nonsynonymous | 25 | 0,9680612 | VUS | HQ2 |
| CLEC18B | SNP | 16 | 74421404 | 74421404 | A | G | rs3851723 | NM_001385192.1 | | c.-127-7T>C | 2 | Homozygous | Splice Region | Other | 34 | 0,7356673 | VUS | HQ2 |
| PLIN4 | SNP | 19 | 4511338 | 4511338 | T | A | rs7259721 | NM_001367868.2 | p.Lys874Asn | c.2622A>T | 5 | Homozygous | Exonic | nonsynonymous | 20 | 0,71064556 | VUS | HQ2 |
| SCAT1 | INDEL | 17 | 78624318 | 78624318 | G | GAAAAAA | rs5822261 | NR_110849.1 | | n.586-9_586-4dup | 4 | Homozygous | Splice Region | Other | 20 | 0,27847606 | VUS | HQ2 |
| PTGES3L | INDEL | 17 | 42969193 | 42969194 | GC | G | rs113173233 | NM_001261430.2 | | c.433-8del | 7 | Homozygous | Splice Region | Other | 25 | 0,661908 | VUS | HQ2 |
| MT-ND5 | SNP | M | 12662 | 12662 | A | G | rs879105366 | ENST00000361567.2 | p.Asn109Ser | c.326A>G | 1 | Homozygous | Exonic | nonsynonymous | 265 | 0,000336676 | VUS | HQ2 |
| PRAMEF10 | SNP | 1 | 12893335 | 12893335 | T | G | rs2637920 | NM_001039361.4 | p.Asn336His | c.1006A>C | 4 | Homozygous | Exonic | nonsynonymous | 28 | 0,48626938 | VUS | HQ2 |
| CHD8 | SNP | 14 | 21392713 | 21392713 | C | T | rs367894618 | NM_001170629.2 | p.Gly2189Arg | c.6565G>A | 34 | Homozygous | Exonic | nonsynonymous | 27 | 1,31461E-05 | Possibly pathogenic - Moderate | HQ2 |
| CYP3A5 | SNP | 7 | 99672916 | 99672916 | T | C | rs776746 | NM_001291829.2 | | c.-253-1A>G | 4 | Homozygous | Splice Acceptor | Other | 21 | 0,72857463 | Possibly pathogenic - Low | HQ2 |
| OR10G2 | SNP | 14 | 21634436 | 21634436 | C | T | rs10138694 | NM_001005466.2 | p.Arg136His | c.407G>A | 1 | Homozygous | Exonic | nonsynonymous | 26 | 0,4835136 | VUS | HQ2 |
| A4GNT | SNP | 3 | 138131004 | 138131004 | A | C | | NM_016161.3 | p.Phe85Val | c.253T>G | 2 | Homozygous | Exonic | nonsynonymous | 22 | | | HQ3 |
| ZNF717 | SNP | 3 | 75737602 | 75737602 | C | T | rs3009020 | NM_001290208.3 | p.Arg674His | c.2021G>A | 5 | Homozygous | Exonic | nonsynonymous | 31 | 0,577362 | VUS | HQ4 |
| PLIN4 | SNP | 19 | 4511915 | 4511915 | G | A | rs10422025 | NM_001367868.2 | p.Ala682Val | c.2045C>T | 5 | Homozygous | Exonic | nonsynonymous | 49 | 0,589142 | VUS | HQ4 |
| RHD | SNP | 1 | 25272669 | 25272669 | A | C | | NM_016124.6 | p.Gln41Pro | c.122A>C | 1 | Homozygous | Exonic | nonsynonymous | 22 | | VUS | HQ4 |
| PLIN4 | SNP | 19 | 4511338 | 4511338 | T | A | rs7259721 | NM_001367868.2 | p.Lys874Asn | c.2622A>T | 5 | Homozygous | Exonic | nonsynonymous | 42 | 0,71064556 | VUS | HQ4 |
| ZNF717 | SNP | 3 | 75737791 | 75737791 | C | A | rs2918517 | NM_001290208.3 | p.Arg611Ile | c.1832G>T | 5 | Homozygous | Exonic | nonsynonymous | 31 | 0,3450476 | VUS | HQ4 |
| ANKRD36C | SNP | 2 | 95960544 | 95960544 | C | T | rs4362598 | NM_001393982.1 | p.Gly311Asp | c.932G>A | 10 | Homozygous | Exonic | nonsynonymous | 36 | 0,9680612 | VUS | HQ4 |
| NPIPB15 | SNP | 16 | 74391650 | 74391650 | A | G | rs2868591 | NM_001306094.2 | p.Tyr301Cys | c.902A>G | 8 | Homozygous | Exonic | nonsynonymous | 28 | 0,6321651 | VUS | HQ4 |
| OR10G2 | SNP | 14 | 21634436 | 21634436 | C | T | rs10138694 | NM_001005466.2 | p.Arg136His | c.407G>A | 1 | Homozygous | Exonic | nonsynonymous | 29 | 0,4835136 | VUS | HQ4 |
| ZNF717 | SNP | 3 | 75738575 | 75738575 | G | A | rs1962893 | NM_001290208.3 | p.Arg350Cys | c.1048C>T | 5 | Homozygous | Exonic | nonsynonymous | 31 | 0,57684606 | VUS | HQ4 |
| NPIPB15 | SNP | 16 | 74391671 | 74391671 | C | T | rs11641596 | NM_001306094.2 | p.Pro308Leu | c.923C>T | 8 | Homozygous | Exonic | nonsynonymous | 30 | 0,4371062 | VUS | HQ4 |
| PROCA1 | SNP | 17 | 28703797 | 28703797 | C | T | rs143950518 | NM_001366301.1 | p.Ala286Thr | c.856G>A | 5 | Homozygous | Exonic | nonsynonymous | 31 | 0,000958408 | VUS | HQ5 |
| KIAA0100 | SNP | 17 | 28634534 | 28634534 | T | C | rs148988447 | NM_014680.5 | p.Asn1018Ser | c.3053A>G | 16 | Homozygous | Exonic | nonsynonymous | 32 | 0,001162501 | VUS | HQ5 |
| ZNF717 | SNP | 3 | 75737602 | 75737602 | C | T | rs3009020 | NM_001290208.3 | p.Arg674His | c.2021G>A | 5 | Homozygous | Exonic | nonsynonymous | 30 | 0,577362 | VUS | HQ5 |
| SIRPA | INDEL | 20 | 1915304 | 1915306 | CCT | C | rs749337996 | NM_001040023.2 | p.Leu96Hisfs*14 | c.287_288del | 2 | Homozygous | Exonic | frameshift | 22 | 0,3653139 | Possibly pathogenic - Moderate | HQ5 |

| | | | | | | | | | | | | | | | | | | |
|---|---|---|---|---|---|---|---|---|---|---|---|---|---|---|---|---|---|---|
| **ZNF717** | SNP | 3 | 75737791 | 75737791 | C | A | rs2918517 | NM_001290208.3 | p.Arg611Ile | c.1832G>T | 5 | Homozygous | Exonic | nonsynonymous | 40 | 0,3450476 | VUS | HQ5 |
| **SIRPA** | INDEL | 20 | 1915303 | 1915303 | A | AGT | rs148409797 | NM_001040023.2 | p.Asp95Glufs*29 | c.284_285insGT | 2 | Homozygous | Exonic | frameshift | 22 | 0,3649683 | Possibly pathogenic - Moderate | HQ5 |
| **OR10G2** | SNP | 14 | 21634436 | 21634436 | C | T | rs10138694 | NM_001005466.2 | p.Arg136His | c.407G>A | 1 | Homozygous | Exonic | nonsynonymous | 26 | 0,4835136 | VUS | HQ5 |
| **ZNF717** | SNP | 3 | 75738575 | 75738575 | G | A | rs1962893 | NM_001290208.3 | p.Arg350Cys | c.1048C>T | 5 | Homozygous | Exonic | nonsynonymous | 39 | 0,57684606 | VUS | HQ5 |
| **ADAP2** | SNP | 17 | 30957856 | 30957856 | G | A | rs142690706 | NM_018404.3 | p.Arg378His | c.1133G>A | 11 | Homozygous | Exonic | nonsynonymous | 23 | 0,001181831 | VUS | HQ5 |
| **PNPLA3** | SNP | 22 | 43934620 | 43934620 | A | G | rs202103609 | NM_025225.3 | p.Ile237Met | c.711A>G | 5 | Homozygous | Exonic | nonsynonymous | 21 | 0,000144568 | Possibly pathogenic - Low | HQ5 |
| **CHRNA5** | SNP | 15 | 78590583 | 78590583 | G | A | rs16969968 | NM_000745.4 | p.Asp398Asn | c.1192G>A | 5 | Homozygous | Exonic | nonsynonymous | 24 | 0,2402897 | VUS | HQ5 |
| **NPIPB15** | SNP | 16 | 74391650 | 74391650 | A | G | rs2868591 | NM_001306094.2 | p.Tyr301Cys | c.902A>G | 8 | Homozygous | Exonic | nonsynonymous | 32 | 0,6321651 | VUS | HQ5 |
| **GLS2** | SNP | 12 | 56471555 | 56471555 | G | A | rs115701548 | NM_013267.4 | p.Leu581Phe | c.1741C>T | 18 | Homozygous | Exonic | nonsynonymous | 23 | 0,000518597 | Possibly pathogenic - Low | HQ5 |
| **NUP205** | SNP | 7 | 135606778 | 135606778 | C | T | rs140772825 | NM_015135.3 | p.Ala978Val | c.2933C>T | 21 | Homozygous | Exonic | nonsynonymous | 52 | 0,000407123 | VUS | HQ5 |
| **NPIPB15** | SNP | 16 | 74391650 | 74391650 | A | G | rs2868591 | NM_001306094.2 | p.Tyr301Cys | c.902A>G | 8 | Homozygous | Exonic | nonsynonymous | 39 | 0,6321651 | VUS | HQ6 |
| **ZNF717** | SNP | 3 | 75738575 | 75738575 | G | A | rs1962893 | NM_001290208.3 | p.Arg350Cys | c.1048C>T | 5 | Homozygous | Exonic | nonsynonymous | 29 | 0,57684606 | VUS | HQ6 |
| **ZNF717** | INDEL | 3 | 75737666 | 75737675 | CACATTCATT | C | rs57577072 | NM_001290208.3 | p.Asn650_Cys652del | c.1948_1956del | 5 | Homozygous | Exonic | nonframeshift | 23 | 0,34304875 | VUS | HQ6 |
| **ZNF717** | SNP | 3 | 75737230 | 75737230 | T | C | rs3009024 | NM_001290208.3 | p.Lys798Arg | c.2393A>G | 5 | Homozygous | Exonic | nonsynonymous | 27 | 0,479239 | VUS | HQ6 |
| **ZNF717** | SNP | 3 | 75737602 | 75737602 | C | T | rs3009020 | NM_001290208.3 | p.Arg674His | c.2021G>A | 5 | Homozygous | Exonic | nonsynonymous | 20 | 0,577362 | VUS | HQ6 |

| Gene | Variation Type | Chr | Start Position | Stop Position | Ref | Alt | dbSNP | Transcript | AA Change | Nucleotide | Exon | Zygosity | Region | Effect | Depth | gnomAD (Genome) | Genoox Classification | Source_file |
|---|---|---|---|---|---|---|---|---|---|---|---|---|---|---|---|---|---|---|
| **NPIPB15** | SNP | 16 | 74391460 | 74391460 | G | A | rs6564065 | NM_001306094.2 | p.Ala238Thr | c.712G>A | 8 | Homozygous | Exonic | nonsynonymous | 33 | 0,54744077 | VUS | PQ1 |
| **FCGBP** | SNP | 19 | 39877755 | 39877755 | C | T | rs28472548 | NM_003890.2 | p.Arg4318His | c.12953G>A | 24 | Homozygous | Exonic | nonsynonymous | 22 | 0,6009629 | VUS | PQ1 |
| **ZNF717** | SNP | 3 | 75737230 | 75737230 | T | C | rs3009024 | NM_001290208.3 | p.Lys798Arg | c.2393A>G | 5 | Homozygous | Exonic | nonsynonymous | 52 | 0,479239 | VUS | PQ1 |
| **NBPF8** | SNP | 1 | 120449388 | 120449388 | G | A | rs1553248447 | NM_001037501.5 | p.Arg325Gln | c.974G>A | 9 | Homozygous | Exonic | nonsynonymous | 50 | 0,6197496 | VUS | PQ1 |
| **NPIPB15** | SNP | 16 | 74391650 | 74391650 | A | G | rs2868591 | NM_001306094.2 | p.Tyr301Cys | c.902A>G | 8 | Homozygous | Exonic | nonsynonymous | 34 | 0,6321651 | VUS | PQ1 |
| **PLIN4** | SNP | 19 | 4511915 | 4511915 | G | A | rs10422025 | NM_001367868.2 | p.Ala682Val | c.2045C>T | 5 | Homozygous | Exonic | nonsynonymous | 49 | 0,589142 | VUS | PQ2 |
| **PRAMEF10** | SNP | 1 | 12893335 | 12893335 | T | G | rs2637920 | NM_001039361.4 | p.Asn336His | c.1006A>C | 4 | Homozygous | Exonic | nonsynonymous | 29 | 0,48626938 | VUS | PQ2 |
| **RGPD3** | SNP | 2 | 106457045 | 106457045 | C | T | rs62152530 | NM_001144013.2 | p.Asp111Asn | c.331G>A | 4 | Homozygous | Exonic | nonsynonymous | 33 | 0,4978853 | VUS | PQ2 |
| **ANKRD36C** | SNP | 2 | 95960544 | 95960544 | C | T | rs4362598 | NM_001393982.1 | p.Gly311Asp | c.932G>A | 10 | Homozygous | Exonic | nonsynonymous | 27 | 0,9680612 | VUS | PQ2 |
| **ANKRD36C** | SNP | 2 | 95960544 | 95960544 | C | T | rs4362598 | NM_001393982.1 | p.Gly311Asp | c.932G>A | 10 | Homozygous | Exonic | nonsynonymous | 20 | 0,9680612 | VUS | PQ3 |
| **POTEJ** | SNP | 2 | 130656603 | 130656603 | C | T | rs62165276 | NM_001277083.2 | p.Arg615Trp | c.1843C>T | 15 | Homozygous | Exonic | nonsynonymous | 26 | 0,50376934 | VUS | PQ3 |
| **A4GNT** | SNP | 3 | 138131004 | 138131004 | A | C | | NM_016161.3 | p.Phe85Val | c.253T>G | 2 | Homozygous | Exonic | nonsynonymous | 41 | | VUS | PQ3 |
| **MUC3A** | SNP | 7 | 100956404 | 100956404 | C | A | rs1367884493 | NM_005960.2 | p.Pro1542His | c.4625C>A | 2 | Homozygous | Exonic | nonsynonymous | 43 | 0,5032913 | VUS | PQ4 |
| **ZNF717** | SNP | 3 | 75738575 | 75738575 | G | A | rs1962893 | NM_001290208.3 | p.Arg350Cys | c.1048C>T | 5 | Homozygous | Exonic | nonsynonymous | 46 | 0,57684606 | VUS | PQ4 |
| **CNTNAP3B** | SNP | 9 | 41953308 | 41953308 | A | C | rs62536501 | NM_001201380.3 | p.Leu652Arg | c.1955T>G | 13 | Homozygous | Exonic | nonsynonymous | 24 | 0,6463537 | VUS | PQ4 |
| **ZNF717** | SNP | 3 | 75737602 | 75737602 | C | T | rs3009020 | NM_001290208.3 | p.Arg674His | c.2021G>A | 5 | Homozygous | Exonic | nonsynonymous | 24 | 0,577362 | VUS | PQ4 |
| **PLIN4** | SNP | 19 | 4511915 | 4511915 | G | A | rs10422025 | NM_001367868.2 | p.Ala682Val | c.2045C>T | 5 | Homozygous | Exonic | nonsynonymous | 44 | 0,589142 | VUS | PQ4 |
| **NPIPB15** | SNP | 16 | 74391650 | 74391650 | A | G | rs2868591 | NM_001306094.2 | p.Tyr301Cys | c.902A>G | 8 | Homozygous | Exonic | nonsynonymous | 22 | 0,6321651 | VUS | PQ4 |
| **NPIPB15** | SNP | 16 | 74391671 | 74391671 | C | T | rs11641596 | NM_001306094.2 | p.Pro308Leu | c.923C>T | 8 | Homozygous | Exonic | nonsynonymous | 25 | 0,4371062 | VUS | PQ4 |
| **PLIN4** | SNP | 19 | 4511338 | 4511338 | T | A | rs7259721 | NM_001367868.2 | p.Lys874Asn | c.2622A>T | 5 | Homozygous | Exonic | nonsynonymous | 32 | 0,71064556 | VUS | PQ4 |
| **FCGBP** | SNP | 19 | 39877858 | 39877858 | A | C | rs12610683 | NM_003890.2 | p.Ser4284Ala | c.12850T>G | 24 | Homozygous | Exonic | nonsynonymous | 21 | 0,9993551 | VUS | PQ4 |
| **ZNF717** | SNP | 3 | 75737791 | 75737791 | C | A | rs2918517 | NM_001290208.3 | p.Arg611Ile | c.1832G>T | 5 | Homozygous | Exonic | nonsynonymous | 37 | 0,3450476 | VUS | PQ4 |
| **AMY2B** | SNP | 1 | 103575270 | 103575270 | T | C | rs140209167 | NM_001387437.1 | p.Val309Ala | c.926T>C | 6 | Homozygous | Exonic | nonsynonymous | 22 | 0,001977557 | VUS | PQ5 |
| **KIAA0100** | SNP | 17 | 28634534 | 28634534 | T | C | rs148988447 | NM_014680.5 | p.Asn1018Ser | c.3053A>G | 16 | Homozygous | Exonic | nonsynonymous | 28 | 0,001162501 | VUS | PQ5 |
| **NUP205** | SNP | 7 | 135606778 | 135606778 | C | T | rs140772825 | NM_015135.3 | p.Ala978Val | c.2933C>T | 21 | Homozygous | Exonic | nonsynonymous | 38 | 0,000407123 | VUS | PQ5 |

| | | | | | | | | | | | | | | | | | | | |
|---|---|---|---|---|---|---|---|---|---|---|---|---|---|---|---|---|---|---|---|
| **SIRPA** | INDEL | 20 | 1915303 | 1915303 | A | AGT | rs148409797 | NM_001040023.2 | p.Asp95Glufs*29 | c.284_285insGT | 2 | Homozygous | Exonic | frameshift | 25 | 0,3649683 | Possibly pathogenic - Moderate | PQ5 |
| **PROCA1** | SNP | 17 | 28703797 | 28703797 | C | T | rs143950518 | NM_001366301.1 | p.Ala286Thr | c.856G>A | 5 | Homozygous | Exonic | nonsynonymous | 27 | 0,000958408 | VUS | PQ5 |
| **SIRPA** | INDEL | 20 | 1915304 | 1915306 | CCT | C | rs749337996 | NM_001040023.2 | p.Leu96Hisfs*14 | c.287_288del | 2 | Homozygous | Exonic | frameshift | 25 | 0,3653139 | Possibly pathogenic - Moderate | PQ5 |
| **ZNF717** | SNP | 3 | 75738575 | 75738575 | G | A | rs1962893 | NM_001290208.3 | p.Arg350Cys | c.1048C>T | 5 | Homozygous | Exonic | nonsynonymous | 47 | 0,57684606 | VUS | PQ5 |
| **ZNF717** | SNP | 3 | 75737791 | 75737791 | C | A | rs2918517 | NM_001290208.3 | p.Arg611Ile | c.1832G>T | 5 | Homozygous | Exonic | nonsynonymous | 25 | 0,3450476 | VUS | PQ5 |
| **PNPLA3** | SNP | 22 | 43934620 | 43934620 | A | G | rs202103609 | NM_025225.3 | p.Ile237Met | c.711A>G | 5 | Homozygous | Exonic | nonsynonymous | 28 | 0,000144568 | Possibly pathogenic - Low | PQ5 |
| **ZNF717** | SNP | 3 | 75737602 | 75737602 | C | T | rs3009020 | NM_001290208.3 | p.Arg674His | c.2021G>A | 5 | Homozygous | Exonic | nonsynonymous | 30 | 0,577362 | VUS | PQ5 |
| **NPIPB15** | SNP | 16 | 74391650 | 74391650 | A | G | rs2868591 | NM_001306094.2 | p.Tyr301Cys | c.902A>G | 8 | Homozygous | Exonic | nonsynonymous | 28 | 0,6321651 | VUS | PQ5 |
| **ZNF717** | SNP | 3 | 75738575 | 75738575 | G | A | rs1962893 | NM_001290208.3 | p.Arg350Cys | c.1048C>T | 5 | Homozygous | Exonic | nonsynonymous | 39 | 0,57684606 | VUS | PQ6 |
| **NPIPB15** | SNP | 16 | 74391650 | 74391650 | A | G | rs2868591 | NM_001306094.2 | p.Tyr301Cys | c.902A>G | 8 | Homozygous | Exonic | nonsynonymous | 63 | 0,6321651 | VUS | PQ6 |
| **ZNF717** | SNP | 3 | 75737791 | 75737791 | C | A | rs2918517 | NM_001290208.3 | p.Arg611Ile | c.1832G>T | 5 | Homozygous | Exonic | nonsynonymous | 23 | 0,3450476 | VUS | PQ6 |
| **ZNF717** | SNP | 3 | 75737230 | 75737230 | T | C | rs3009024 | NM_001290208.3 | p.Lys798Arg | c.2393A>G | 5 | Homozygous | Exonic | nonsynonymous | 27 | 0,479239 | VUS | PQ6 |
| **ZNF578** | SNP | 19 | 52511694 | 52511694 | A | T | c.1832G>T | NM_001099694.2 | p.His438Leu | c.1313A>T | 6 | Homozygous | Exonic | nonsynonymous | 24 | | VUS | PQ6 |
| **ZNF717** | SNP | 3 | 75737602 | 75737602 | C | T | rs3009020 | NM_001290208.3 | p.Arg674His | c.2021G>A | 5 | Homozygous | Exonic | nonsynonymous | 26 | 0,577362 | VUS | PQ6 |
| **FOXO6** | INDEL | 1 | 41382200 | 41382200 | G | *** | | NM_001291281.3 | p.Arg404Glyfs*106 | *** | 2 | Homozygous | Exonic | frameshift | 22 | 0,9973426 | Possibly pathogenic - Moderate | PQ6 |

*** Alt:
GGCCCGCGCCGGGACGCCCGCCTACTTCGGCGGCTGCAAGGGCGGCGCCTACGGCGGGGGCGGGGGCTTCGGGCCGCCGGCGATGGGCGCTCTGCGCCGTCTGCCCATGCAGACCATCCAGGAGAACAAGCAGGCCAGCTTCGTGCCGGCCGCGGCGCCCTTCCGCCCTGGGGCGCTGCCCGCGCTGCTGCCGCCGCCGCC

*** Nucleotide:
c.1008_1209+1insGGGACGCCCGCCTACTTCGGCGGCTGCAAGGGCGGCGCCTACGGCGGGGGCGGGGGCTTCGGGCCGCCGGCGATGGGCGCTCTGCGCCGTCTGCCCATGCAGACCATCCAGGAGAACAAGCAGGCCAGCTTCGTGCCGGCCGCGGCGCCCTTCCGCCCTGGGGCGCTGCCCGCGCTGCTGCCGCCGCCGCCGCCCGCGCC

This table summarizes all candidate variants detected in high-quality (HQ) and poor-quality (PQ) sperm pools by whole-genome sequencing (WGS). Listed variants include single-nucleotide variants (SNVs) and insertions/deletions (indels) located in exonic regions, splice sites, and splice-region boundaries. For each variant, the table provides the gene name, variant class, chromosomal position, reference and alternate alleles, dbSNP identifier (when available), transcript annotation, amino acid and nucleotide changes, exon location, inferred zygosity in the corresponding individual, genomic region, predicted functional effect, sequencing read depth, and gnomAD genome allele frequency. Variant classifications were assigned using the Franklin (Genoox) platform according to ACMG criteria. The “Source_file” column indicates whether the variant was detected in an HQ or PQ sperm pool. Because sequencing was performed on pooled DNA derived from individually selected spermatozoa, zygosity was interpreted only at the level of the corresponding individual and not at the level of individual sperm cells.

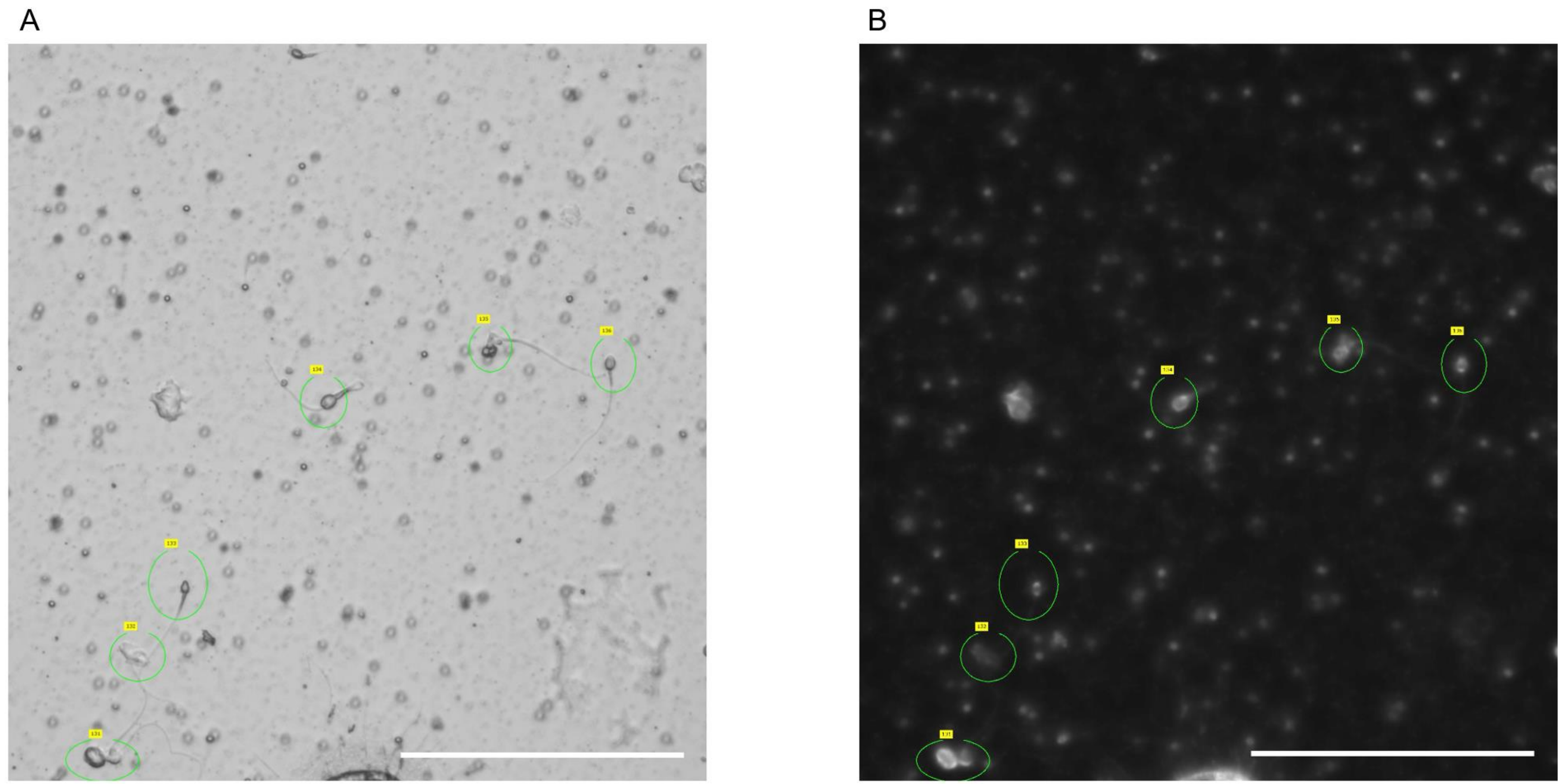


**Supplemental Figure 1.** Single Sperm Selection for PALM® MicroBeam Microdissection System.

Individual sperm were identified for microdissection using phase-contrast imaging (A) followed by fluorescence detection of DAPI-stained nuclear DNA (B). Green circles with yellow numbers indicate the sperm selected for isolation. Scale bars: 75 µm.
